# Versatile selective evolutionary pressure using synthetic defect in universal metabolism

**DOI:** 10.1038/s41467-021-27266-9

**Published:** 2021-11-25

**Authors:** Lara Sellés Vidal, James W. Murray, John T. Heap

**Affiliations:** 1 Imperial College Centre for Synthetic Biology, Imperial College London, London, SW7 2AZ UK; 2 Department of Life Sciences, Imperial College London, London, SW7 2AZ UK; 3 School of Life Sciences, The University of Nottingham, Biodiscovery Institute, University Park, Nottingham, NG7 2RD UK

**Keywords:** Biocatalysis, Protein engineering, Metabolic engineering, Synthetic biology, Protein design

## Abstract

The non-natural needs of industrial applications often require new or improved enzymes. The structures and properties of enzymes are difficult to predict or design *de novo*. Instead, semi-rational approaches mimicking evolution entail diversification of parent enzymes followed by evaluation of isolated variants. Artificial selection pressures coupling desired enzyme properties to cell growth could overcome this key bottleneck, but are usually narrow in scope. Here we show diverse enzymes using the ubiquitous cofactors nicotinamide adenine dinucleotide (NAD) or nicotinamide adenine dinucleotide phosphate (NADP) can substitute for defective NAD regeneration, representing a very broadly-applicable artificial selection. Inactivation of *Escherichia coli* genes required for anaerobic NAD regeneration causes a conditional growth defect. Cells are rescued by foreign enzymes connected to the metabolic network only via NAD or NADP, but only when their substrates are supplied. Using this principle, alcohol dehydrogenase, imine reductase and nitroreductase variants with desired selectivity modifications, and a high-performing isopropanol metabolic pathway, are isolated from libraries of millions of variants in single-round experiments with typical limited information to guide design.

## Abstract (web summary)

Rational design of enzymes with new or improved properties is rarely straightforward, and artificial selection pressure approaches that link an improvement in the target to cell growth are an alternative. Here, the authors show that diverse enzymes sharing the ubiquitous cofactor NAD(P)^+^ can substitute for defective NAD^+^ regeneration, representing a very broadly-applicable artificial selection.

## Introduction

Catalysts are essential to increase chemical reaction rates in a wide range of industries. Enzymes have advantages over non-biological catalysts including high chemoselectivity, stereoselectivity, and positional selectivity; and activity under mild temperature and pressure conditions, which also decreases undesired side reactions^1^. These advantages can decrease costs and increase process performance and sustainability. Enzymes are used both in industries with unchanging catalytic requirements, such as the manufacture of commodity chemicals and products or as active agents in cleaning products; and to meet the ongoing need for new specialized catalytic steps in synthetic-chemistry routes to bespoke pharmaceutical or agrichemical compounds^2^. However, despite the vast diversity of natural enzymes and the reactions they catalyze, often no optimal enzyme can be identified for a specific application, typically due to low affinity or activity for the target substrate or a lack of stability or activity under required conditions such as high temperatures or organic solvents^3^.

In principle, enzymes with new or improved properties could be designed rationally on the basis of structural information, or even *de novo*, but in practice, this is rarely straightforward. Directed evolution is one of the most powerful tools for protein engineering, since it bypasses the need to determine specific sequences and structures a priori by mimicking the process of natural evolution in the laboratory^2,4^. Starting from one or more parent enzymes with properties close to the desired ones, libraries of many variants are readily generated using targeted and/or random mutagenesis. Variants with desired improvements occur very rarely in such libraries, if at all, so identifying and isolating improved variants is usually the key challenge^5^. Typically, variants of interest are identified through screening, where individual variants from samples of the library are isolated and the target property evaluated. This is generally a laborious and time-consuming process, except in special cases with convenient readouts such as fluorescence^5^. The need for screening is a fundamental constraint on throughput which limits the success and scope of directed evolution. Artificial selection pressure approaches offer an attractive alternative, since they link an improvement in the target property to the survival of the variant. Such approaches do not require variants to be isolated for evaluation and automatically discard non-functional variants, thus allowing much higher throughput than screening. However, the techniques available so far tend to be narrowly applicable only to very specific types of biomolecules or properties^6^. Here, we exploit a synthetic metabolic defect to apply a broadly-applicable artificial selection pressure to diverse biocatalysts, using genetically-modified *E. coli* under special conditions.

**Fig. 1:**
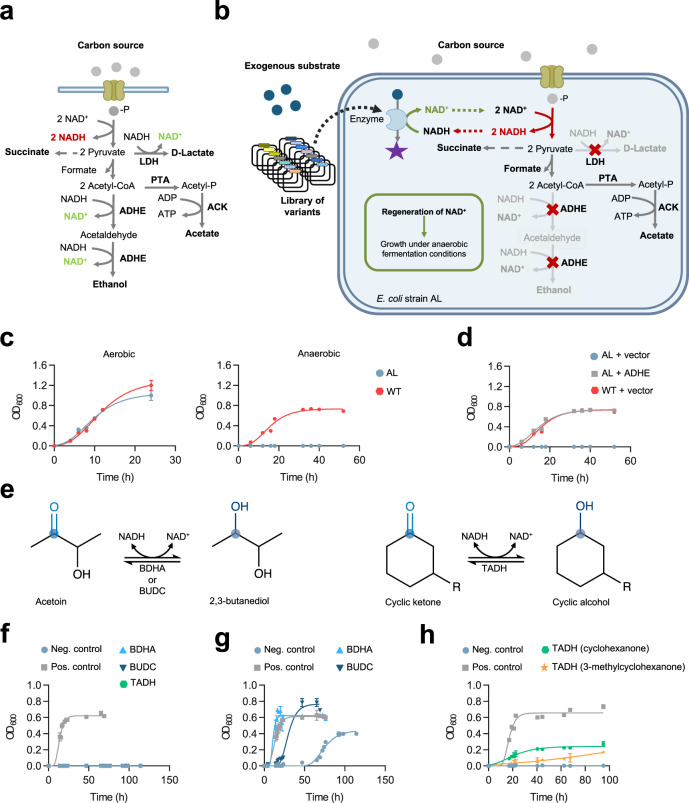
Principle and validation of redox-directed in vivo evolution. **a** *E. coli* anaerobic fermentation pathways. In anaerobic fermentation, reduction of pyruvate to ethanol and/or lactate regenerates NAD^+^ required for glycolysis and growth. **b** *E. coli* strain AL lacking ADHE (bifunctional alcohol-aldehyde dehydrogenase) and LDH (lactate dehydrogenase) cannot regenerate NAD^+^, so cannot grow by anaerobic fermentation. Under anaerobic fermentation conditions, selection pressure to restore NAD^+^ regeneration and hence growth can be used to select variants from libraries, such as enzyme variants active with non-natural substrates. **c** Cultures of WT and AL cells in M9 glucose medium under aerobic or anaerobic conditions. **d** Transformation with ADHE (pLS1, hereafter ‘Pos. control’) but not vector (pUC19, hereafter ‘Neg. control’) restores anaerobic growth of AL cells in M9 glucose to WT levels. **e** Foreign enzyme-substrate pairs used as proof of concept of the selection system. **f** AL cells transformed with BDHA (*Bacillus subtilis* acetoin reductase; pLS2), BUDC (*Klebsiella pneumoniae* acetoin reductase; pLS3), or TADH (*Thermus* sp. ATN1 alcohol dehydrogenase; pLS12) do not grow anaerobically in M9 glucose medium without the oxidized substrate of each oxidoreductase. Addition of acetoin (**g**) or cyclohexanone or 3-methylcyclohexanone (**h**) to the culture medium restored anaerobic growth in M9 glucose medium of AL cells transformed with BDHA or BUDC (**g**) or TADH (**h**). Data points of growth curves represent mean values, with error bars showing standard deviation; *n* = 3 biologically independent cultures for all timepoints of growth curves. Source data are provided as a Source Data file.

Wild-type (WT) *E. coli* is a facultative anaerobe, normally able to grow by aerobic respiration using oxygen, anaerobic respiration using alternative electron acceptors, or anaerobic fermentation without an external electron acceptor. Strict anaerobic fermentation is a closed system in which consumption by glycolysis of a finite supply of the redox cofactor oxidized nicotinamide adenine dinucleotide (NAD^+^) must be stoichiometrically matched by regeneration of NAD^+^ through reduction of central metabolites to form fermentation products. In *E. coli*, NAD^+^ is normally regenerated largely by ethanol formation. Unlike in yeast, ethanol production serves no other known physiological role in *E. coli*. Inactivation of this pathway prevents *E. coli* growth by anaerobic fermentation^7^ (Fig. 1a), presumably due mainly to NAD^+^ depletion blocking glycolysis, although pyruvate and acetyl-CoA will also accumulate to unnatural levels with various impacts on metabolism. Anaerobic fermentative growth of such mutants can be rescued by introducing pathways that reinstate fluxes from central metabolites and restore NAD^+^ regeneration. This can provide a useful selection pressure to identify redox-active pathways, enzymes, and variants with desired properties useful for the production of products such as butanol, 2-methylpropan-1-ol, L-alanine, and 2,3-butanediol^8–11^ (related selections can be achieved involving different mutations, NADP and/or aerobic conditions^12–14^). It is difficult to discern the contributions to the selection of NAD^+^ regeneration, relieving central metabolite accumulation, and restoring other fluxes.

We hypothesized that regeneration of NAD^+^ by a metabolically isolated foreign enzyme, using an externally-supplied substrate not otherwise connected to the native metabolic network, would be sufficient to restore glycolysis and thereby growth. If so, a strong artificial selection pressure could be applied to libraries of enzyme variants for activity with substrates that are not produced within cells, linking restoration of growth by anaerobic fermentation to the presence of a variant possessing a desired new or improved activity with a non-natural substrate (Fig. 1b). This broader scope would be compatible with the development of biocatalysts active upon non-biogenic compounds for which no biosynthetic pathway exists in any organism, so cannot be produced using introduced heterologous pathways, which represent the great majority of chemotypes used as precursors and intermediates in synthetic chemistry. Reports published during the present study applied such selection using acetoin^11,13^, but *E. coli* contains native acetoin reductase activity^11,15^, and our present study found acetoin alone was sufficient to rescue the metabolic defect, so acetoin cannot be considered isolated from native *E. coli* metabolism.

Here we validate and expand upon the above concept, apply it to rapidly obtain variants from large libraries (comprising tens of millions of variants) in several different cases, and hence establish a rapid, high-throughput, widely-applicable platform for directed evolution of biocatalysts and other biomolecules whose activity can be directly or indirectly coupled to the regeneration of NAD^+^.

## Results

### Synthetic defects in NAD^+^ regeneration abolishing anaerobic growth can be rescued by metabolically isolated foreign enzyme-substrate pairs

To implement the artificial-selection system, a genetically-modified *E. coli* strain (strain AL) unable to regenerate NAD^+^ during anaerobic fermentation was constructed by inactivating the genes encoding the ethanol pathway (bifunctional aldehyde-alcohol dehydrogenase, *adhE*) and, to eliminate a potential mutational escape route^7^, the lactate pathway (lactate dehydrogenase, *ldhA*). When cultured aerobically in a minimal medium, AL cells grew similarly to WT cells. However, they were unable to grow anaerobically in the same medium, indicating the expected impairment of anaerobic fermentation (Fig. 1c). We validated this strain by genetic complementation, through a transformation with a plasmid bearing the WT *adhE* gene, which allowed anaerobic growth similar to WT cells, reaching a maximum optical density at 600 nm (OD_600_) of 0.8 by 24−36 h (Fig. 1d). ^1^H**-**NMR spectra of the fermentation broth confirmed the profile of metabolites was similar to that of the parental strain (except for the expected absence of lactate, Supplementary Table 1).

We attempted to restore anaerobic growth of AL cells using exogenous enzyme-substrate pairs. In each case, the substrate was not expected to be natively produced or consumed by *E. coli*, so the main link to the *E. coli* metabolic network was through NAD. Three NADH-dependent oxidoreductases were tested: acetoin reductases from *Bacillus subtilis* (BDHA)^16^ and *Klebsiella pneumoniae* (BUDC)^17^, and alcohol dehydrogenase from *Thermus* sp. ATN1 (TADH)^18^ (Fig. 1e). AL cells transformed with any of the exogenous reductases were able to grow anaerobically in minimal medium only if also supplemented with the corresponding substrate (acetoin for BDHA and BUDC, and cyclohexanone or 3-methylcyclohexanone for TADH) (Fig. 1f−h). The substrates alone did not restore growth except for acetoin, with which growth was eventually observed, probably due to the activity of an endogenous acetoin reductase^11,15^. The consumption of substrates and generation of corresponding reduced products was confirmed in all three cases using ^1^H-NMR (Supplementary Table 1). These results with cyclohexanone and 3-methylcyclohexanone indicate that NAD^+^ regeneration alone, without any effects of carbon flux with metabolism, is enough to rescue AL cells from their metabolic defect.

### High-throughput artificial selection outperforms computational design of cofactor specificity reversal

NAD(P)-dependent oxidoreductases have been extensively engineered to make them more suitable for specific applications, targeting properties such as kinetic parameters, substrate specificity, or cofactor preference^19–22^. Switching the cofactor preference of NADP-dependent enzymes to NAD is of particular interest due to the lower cost and higher stability of NAD, and the higher efficiency of cell-free NAD recycling systems^23,24^, for which low-cost co-substrates are available. Substrate selectivities, and modifications to them, are not generally computationally predictable. NAD/NADP selectivity is a partial exception, as the many known examples and a common structural motif enabled the development of a computational design tool, CSR-SALAD, which represents the state of the art in cofactor-selectivity reversal^25,26^.

**Fig. 2:**
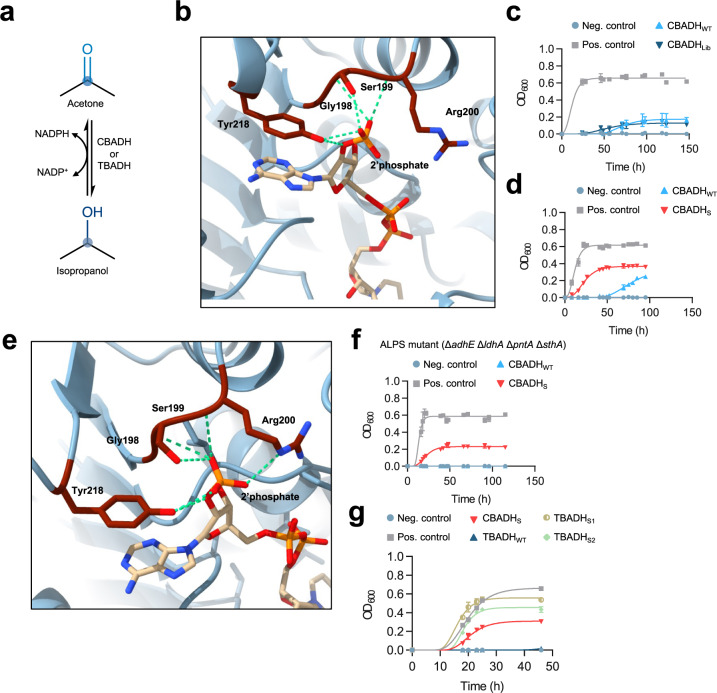
Selection of alcohol dehydrogenases with reversed cofactor selectivity. **a** CBADH (*Clostridium beijerinckii* alcohol dehydrogenase) and TBADH (*Thermoanaerobacter brockii* alcohol dehydrogenase) catalyze the reduction of acetone to isopropanol exclusively using NADPH, as well as the reverse reaction. **b** Cofactor binding site of CBADH_WT_ with NADPH bound (PDB code 1KEV). The S199, R200, and Y218 residues interact with the 2′ phosphate of NADPH. Hydrogen bonds are shown in green. **c** Anaerobic culture of, AL cells transformed with library CBADH_Lib_ (pLS10) in M9 glucose medium supplemented with acetone. Controls are as in Fig. 1. CBADH_WT_ (pLS6) was able to restore the slow anaerobic growth of AL cells due to the activity of transhydrogenases. **d** Anaerobic growth of AL cells transformed with CBADH_S_ (with substitutions G198D, S199Y, and Y218P; pLS10_3) in medium with acetone. **e** Cofactor binding site of TBADH_WT_ with NADPH bound (PDB code 1YKF). A similar set of residues as in CBADH establishes interactions with the 2′ phosphate of TBADH. Hydrogen bonds are shown in green. **f** Anaerobic culture of ALPS cells in medium supplemented with acetone. CBADH_S_, which can use NAD, supported anaerobic growth of ALPS. Unlike AL cells transformed with CBADH_WT_, ALPS cells transformed with CBADH_WT_ did not grow anaerobically, due to the absence of any transhydrogenase activity to regenerate NAD^+^ from NADP^+^. **g** Anaerobic culture of ALPS cells transformed with CBADH_S_, TBADH_S1_ (with substitutions G198S, S199K, R200P, and Y218V; pLS73_2), TBADH_S2_ (with a duplication of residues 191−241 and substitutions G198H, S199R, R200A, Y218M, G198’A, and R200’K; pLS73_1) or TBADH_WT_ (pLS69) in medium supplemented with acetone. All NAD-utilizing variants supported the anaerobic growth of ALPS. TBADH_WT_, like CBADH_WT_, could not support the anaerobic growth of ALPS cells. Data points of growth curves represent mean values, with error bars showing standard deviation; *n* = 3 biologically independent cultures for all timepoints of growth curves. Source data are provided as a Source Data file.

Alcohol dehydrogenases (ADHs), also known as ketoreductases (KREDs), are of great industrial interest primarily due to their ability to perform an asymmetric reduction of aldehydes and ketones. Thanks to their regioselectivity and stereoselectivity, ADHs can be used not only to produce enantiomerically pure alcohols, but also other types of compounds, such as γ- and δ-lactones through the desymmetrisation of *meso*-diols^27,28^. The primary-secondary alcohol dehydrogenase of *Clostridium beijerinckii* (CBADH) reduces this organism’s waste acetone to isopropanol, but is strictly NADP-dependent, which is unusual in anaerobic fermentation^29^ (Fig. 2a). An efficient NAD-utilizing acetone reductase variant of CBADH would be useful in industrial biotechnology and cofactor recycling, so we set out to evolve one.

CSR-SALAD was used to analyze CBADH, predict residues critical for cofactor preference, and design a cofactor preference reversal strategy. The software suggested 567 protein variants encoded by a library of 648 genetic variants of positions G198 (substitutions to DEGKNRS), S199 (to DGHILNRSV), and Y218 (to ADFINSTVY), located at the cofactor binding pocket close to the 2′ phosphate group of NADP (Fig. 2b). CSR-SALAD assumes reversal would cause substantial loss of activity, and proposed subsequent rounds of site-saturation mutagenesis and screening to attempt to recover activity, initially prioritizing I175 and R200. A previous manual analysis of the CBADH crystal structure proposed substitutions of residues G198, S199, R200, and Y218 to reverse cofactor preference^29^, but neither the specific substitutions proposed nor saturation mutagenesis of S199 yielded any NADH-dependent variants of the closely-related *Clostridium autoethanogenum* ADH^30^. Here we applied the proposed substitutions to CBADH, constructing variants CBADH_R1_ (S199G, R200G, and Y218F) and CBADH_R2_ (G198D, S199G, R200G, and Y218F), but plasmids encoding these variants did not restore anaerobic growth of AL cells in minimal medium supplemented with 15 mM acetone, indicating low or absent NADH-dependent activity.

We performed full combinatorial saturation mutagenesis of all four target residues of CBADH (G198, S199, R200, and Y218) replacing each codon with NNN to yield a library (CBADH_Lib_) of 16.8 million unique genetic variants encoding 160,000 unique protein variants. Every variant proposed by CSR-SALAD was included, comprising only 0.4% of the library. By including R200, the activity loss that CSR-SALAD envisages as an emergent problem to be solved by subsequent recovery is addressed simultaneously during cofactor preference reversal.

To select for CBADH variants able to accept NAD, AL cells were transformed with the library, and pools of variants were incubated anaerobically in a minimal medium supplemented with 15 mM acetone, so that any variant with NADH-dependent acetone-reducing activity would lead to a regeneration of NAD^+^, restoring growth. For this library and each similar case in this study, multiple independent transformations (six for this library) were used to obtain complete library coverage (approximately 18 million transformant clones in this case) and samples were sequenced to confirm diverse codon incorporation. Clones from each transformation were pooled and subjected to selection separately. Growth was observed in three of the variant pools (Fig. 2c) so these were each subcultured under the same conditions once, then plasmid DNA was extracted and sequenced, revealing the presence of the same CBADH variant in each case (CBADH_S_) with G198D, S199Y, and Y218P substitutions. Interestingly, NAD-dependent mouse class II alcohol dehydrogenase (ADH2)^31^, which is distantly related to CBADH, contains D227 and P247 which are equivalent positions to 198 and 218 in CBADH. CBADH_S_ was not among the 567 variants proposed by CSR-SALAD. The selection of the same variant in three independent experiments indicates the superiority of this variant, the strength of the artificial selection pressure, and its utility to isolate variants. Transforming AL cells with isolated plasmid encoding CBADH_S_ enabled fast anaerobic growth in a medium containing acetone (Fig. 2d). Acetone consumption and corresponding isopropanol generation were confirmed by ^1^H-NMR (Supplementary Table 1). Unlike the parent enzyme CBADH_WT_, purified CBADH_S_ was unable to oxidize isopropanol using oxidized NADP (NADP^+^) as the cofactor, but could do so with NAD^+^ (*K*_m_ = 17.49 mM, *k*_cat_ = 333 min^−1^ and *k*_cat_/K_m_ = 316.67 M^−1^ s^−1^, Supplementary Fig. 1 and Supplementary Table 2).

### Eliminating transhydrogenase activities provides strict cofactor selection allowing exceptional reversal of cofactor preference

Unexpectedly, we observed that AL cells were able to slowly grow anaerobically in a medium with acetone when transformed with the parent enzyme CBADH_WT_, despite its strict NADP-dependence (Fig. 2c, d). We hypothesized that acetone-dependent anaerobic growth recovery by CBADH_WT_ could be mediated by the activity of one or both of *E. coli*’s two transhydrogenases, PNTA and STHA, which could use the NADP^+^ produced by CBADH_WT_ to oxidize NADH. To test this hypothesis, we generated *E. coli* strains with knockout mutations of transhydrogenase genes *pntA* (ALP strain), *sthA* (ALS strain), or both (ALPS strain) in addition to knockout mutations of *adhE* and *ldhA*. We then tested the ability of CBADH_WT_ and CBADH_S_ to restore anaerobic growth of ALP, ALS, and ALPS cells. The ALP and ALS strains, each with one intact transhydrogenase gene, were able to grow anaerobically in a medium with acetone when transformed with either NADP-dependent CBADH_WT_ or NAD-dependent CBADH_S_ (Supplementary Fig. 2). However, only NAD-dependent CBADH_S_ could support acetone-dependent anaerobic growth of ALPS cells, which lack either intact transhydrogenase gene (Fig. 2f and Supplementary Fig. 2). This demonstrates that transhydrogenases are indeed responsible for the acetone-dependent recovery of anaerobic growth by CBADH_WT_, and that either STHA or PNTA alone is sufficient to generate the required NAD^+^ to sustain anaerobic growth. Interestingly, this is thought to be the non-physiological direction for the membrane-bound, ‘energy-linked’ transhydrogenase PntA^32^. Due to its inability to grow anaerobically when transformed with an NADP-dependent oxidoreductase, the transhydrogenase-free ALPS strain provides a more stringent selection system than the AL, ALP, or ALS strains, strictly requiring NAD-dependent oxidoreductase activity to restore growth.

To validate the ALPS strain as a selection host, we used it to evolve NAD-dependent variants of TBADH, an NADP-dependent alcohol dehydrogenase from *Thermoanaerobacter brockii* closely related to CBADH^29^. Similarly to CBADH_WT_, WT TBADH was able to support the anaerobic growth of AL cells in a medium supplemented with acetone, but not of ALPS cells (Fig. 2g). We performed full combinatorial saturation mutagenesis of residues G198, S199, R200 and Y218 of TBADH to generate a library (TBADH_Lib_) of 16.8 million unique genetic variants (Fig. 2e). A similar selection procedure as for CBADH was followed but using the ALPS strain, and two different variants were identified, TBADH_S1_ and TBADH_S2_. TBADH_S1_ had substitutions G198S, S199K, R200P, and Y218V. Surprisingly, TBADH_S2_ contained a duplication of residues 191−241, in addition to substitutions in the targeted residues, both in the positions of the original sequence and in the corresponding positions of the duplication (Supplementary Fig. 3). The substitutions were G198H, S199R, R200A, Y218M, G198’A, and R200’K (198’ and 200’ denote positions 249 and 251 of TBADH_S2_, the positions in the duplication equivalent to the original 198 and 200 residues). Since the duplication was not intentionally introduced into the variant library by design, it presumably arose through a rare event during the PCR, ligation or within cells, highlighting the potential of the selection system to isolate variants with desired properties even when these are rare and outside the intended design space.

TBADH_S1_ and TBADH_S2_ were both able to restore the anaerobic growth of ALPS cells in a medium with acetone (Fig. 2g), and ^1^H-NMR of the fermentation broth confirmed the production of isopropanol (Supplementary Table 1). When the enzymatic activity was assayed, TBADH_S1_ could oxidize isopropanol only with NAD^+^, certifying the reversal of cofactor preference (Supplementary Fig. 1 and Supplementary Table 2). The *k*_cat_ of TBADH_S1_ for the oxidation of isopropanol with NAD^+^ was 112 ± 5.7 min^−1^, 4.5 times lower than the *k*_cat_ of TBADH_WT_ for the same reaction with NADP^+^. However, the *K*_m_ of TBADH_S1_ for isopropanol was 3.74 ± 0.54 mM, a 32-fold decrease (improvement) compared to the *K*_m_ of TBADH_WT_ for isopropanol in the presence of NADP^+^. Overall, the catalytic efficiency (*k*_cat_/*K*_m_) of TBADH_S1_ for the oxidation of isopropanol (*k*_cat_/*K*_m_ = 496.67 M^−1^ s^−1^) was more than seven times greater than that of TBADH_WT_ with NADP^+^ (*k*_cat_/*K*_m_ = 70 M^−1^ s^−1^). This is, to our knowledge, the highest relative catalytic efficiency obtained in any case of cofactor specificity reversal for an alcohol dehydrogenase, and the best for the reversal of preference from NADP to NAD for any enzyme. In contrast, TBADH_S2_ was able to oxidize isopropanol both with NAD^+^ and NADP^+^ (in the presence of NAD^+^, *K*_m_ = 22.07 mM, *k*_cat_ = 238.5 min^−1^ and *k*_cat_/*K*_m_ = 180 M^−1^ s^−1^; in the presence of NADP^+^, *K*_m_ = 55.15 mM, *k*_cat_ = 231.4 min^−1^ and *k*_cat_/*K*_m_ = 70 M^−1^ s^−1^; Supplementary Fig. 1, Supplementary Table 2). A decrease in *k*_cat_ compared to TBADH_WT_ was observed, but the *K*_m_ for isopropanol decreased (improved) in the presence of both cofactors.

**Fig. 3:**
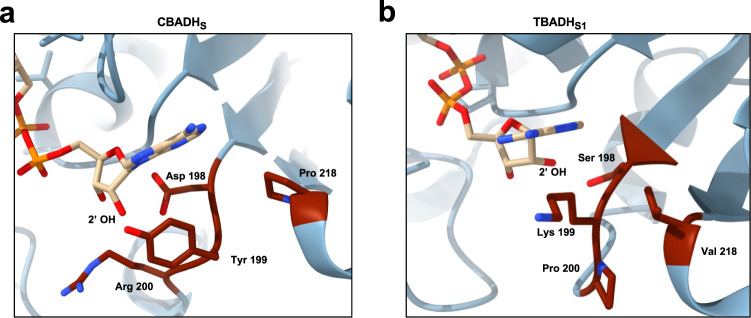
Crystal structures of CBADH_S_ and TBADH_S1_ reveal mechanism of cofactor selectivity reversal. **a** Cofactor binding site of CBADHS with NAD^+^ bound. The substitutions identified in CBADH_S_ allow for the binding of NAD^+^ but would prevent the binding of NADP^+^ by steric impediments and electrostatic repulsion with the side chains of D198 and Y199. Additionally, the stacked-ring interaction of the adenine moiety with Y218 observed in CBADH_WT_ cannot be established due to the Y218P substitution, possibly enabling a more flexible binding of the cofactor. **b** Cofactor binding site of TBADH_S1_, with NAD^+^ placed at the same position as NADP^+^ in the structure of TBADH_WT_. P200−P201 is modelled as a cis peptide bond. While TBADH_S1_ can accommodate NAD^+^ in its cofactor binding pocket, the binding of NADP^+^ would be prevented by steric impediments caused by the side chains of S198 and K199. As in CBADH_S_, the substitution of Y218 prevents the formation of a stacked-ring interaction with the adenine ring of the cofactor, possibly enabling a more flexible binding of NAD^+^.

We crystallized CBADH_S_ and TBADH_S1_ in order to elucidate the structural basis for the observed reversal of cofactor preference. The resulting maps showed clear density for NAD^+^ in the case of CBADH_S_, but only partial occupancy for TBADH_S1_ (Supplementary Fig. 4). In both cases, the size of the cofactor-binding pocket is reduced due to the substitution of residues 198 and 199 by others with bulkier side chains (Fig. 3), which sterically prevents the binding of the 2′ phosphate of NADP. In the case of CBADH_S_, this effect is further enhanced by the presence of an aspartate residue at position 198, which would also prevent NADP binding through electrostatic repulsion between the side-chain carboxylate group and the 2′ phosphate of NADP. Another common feature of both CBADH_S_ and TBADH_S1_ is the substitution of Y218. The side chain of Y218 is known to undergo a 120° rotation in the WT enzymes to allow stacking to the adenine moiety of NADP and the formation of a hydrogen bond with the 2′ phosphate through its hydroxyl group^29^. As shown by the CBADH_S_ and TBADH_S1_ structures, the side chains of the substituted residues are not close enough to the 2′ phosphate to interact with it, and would only be able to form hydrophobic interactions with the adenine moiety, if any (Fig. 3).

### Simultaneous optimization of multiple kinetic parameters by high-throughput artificial selection

**Fig. 4:**
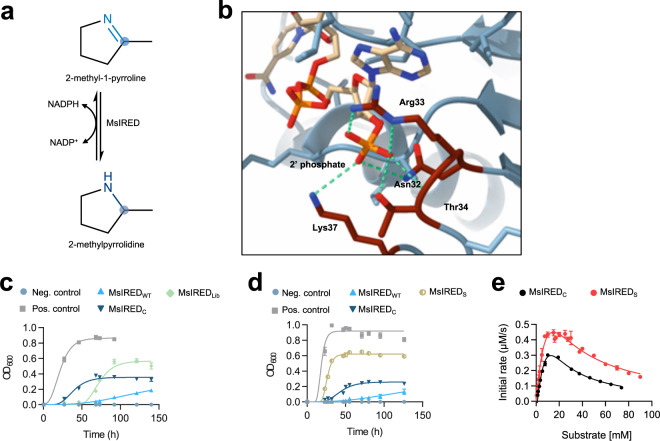
Selection of an imine reductase with reversed cofactor selectivity and minimized substrate inhibition. **a** Reaction catalyzed by MsIRED (*Myxococcus stipitatus* imine reductase). MsIRED can reduce several imines to the corresponding amine using NADPH as cofactor. **b** Homology model of the cofactor binding site of MsIRED_WT_ with NADPH bound. The model was generated by threading the MsIRED sequence into PDB code 3ZHB. Residues 32, 33, 34, and 37 were predicted to be close to the 2’ phosphate of NADPH. Hydrogen bonds are shown in green. **c** Anaerobic culture of AL cells transformed with MsIRED_WT_ (pLS130), MsIRED_C_ (with substitutions N32E, R33Y, T34E, K37R, L67I, and T71V; pLS131) and library MsIRED_Lib_ (pLS133) in medium with 2-methyl-1-pyrroline. Controls are as in Fig. 1. **d** Anaerobic culture of AL cells transformed with MsIRED_WT_, MsIRED_C_, and MsIRED_S_ (with substitutions N32D, R33V, T34R, and K37R) in M9 glucose medium with 2-methyl-1-pyrroline. MsIRED_S_ caused the best anaerobic growth of AL cells. **e** Comparison of enzymatic activity at different concentrations of 2-methyl-1-pyrroline for MsIRED_C_ and MsIRED_S_. Data points of panels **c**−**e** represent mean values, with error bars showing standard deviation. For panels **c** and **d**, *n* = 3 biologically independent cultures for all timepoints of growth curves. For panel **e**, *n* = 3 biologically independent assays for all substrate concentrations with both enzymes. Source data are provided as a Source Data file.

Next, we applied the artificial selection system to an imine reductase (IRED). IREDs are of great industrial interest thanks to their ability to catalyze the asymmetric reduction of imines and the reductive amination of ketones, both of which yield chiral amines, fundamental building blocks in the pharmaceutical and agrichemical industries^33,34^. All known natural IREDs are NADP dependent^35^, so there is great interest in developing NAD-dependent variants due to the lower cost and higher efficiency of NAD-regeneration systems. The most active NAD-dependent IRED thus far is MsIRED_C_, a variant of *Myxococcus stipitatus* IRED (MsIRED), which is able to reduce 2-methyl-1-pyrroline amongst other substrates (Fig. 4a). MsIRED_C_ was obtained through several rounds of mutagenesis and screening^36^. We aimed to obtain a superior NAD-dependent variant of MsIRED through a faster and simpler workflow by applying the artificial selection system.

CSR-SALAD predicted residues N32, R33, T34, and K37 to be critical determinants of cofactor specificity (Fig. 4b). Thus, we performed full combinatorial saturation mutagenesis of these residues to generate a library (MsIRED_Lib_) of 16.8 million unique genetic variants. AL cells transformed with the library were able to grow anaerobically in medium with 2-methyl-1-pyrroline (Fig. 4c), and the same MsIRED variant (MsIRED_S_) was identified from three independent transformations of the library, comprising residue substitutions N32D, R33V, T34R, and K37R. We analyzed the fermentation broth of AL cells transformed with MsIRED_S_ (Fig. 4d) by ^1^H-NMR, confirming the consumption of 2-methyl-1-pyrroline and the production of the corresponding amine, 2-methylpyrrolidine (Supplementary Table 1). The enzymatic activities of MsIRED_C_ and MsIRED_S_ were compared (for MsIRED_C_, *K*_m_ = 34.06 mM, *k*_cat_ = 161.17 min^−1^ and *K*_i_ = 4.94 mM; for MsIRED_S_, *K*_m_ = 19.57 mM, *k*_cat_ = 78.1 min^−1^ and *K*_i_ = 11.42 mM; Supplementary Fig. 5, Supplementary Table 2). Both variants were able to reduce 2-methyl-1-pyrroline only with NADH and showed substrate inhibition, as shown by the decrease in activity at the highest concentrations of substrate. MsIRED_S_ performed better than MsIRED_C_ at all tested substrate concentrations (Fig. 4e), partly due to the higher value of the substrate inhibition constant *K*_i_, which indicates a relief of substrate inhibition in MsIRED_S_ compared to MsIRED_C_. To our knowledge, MsIRED_S_ has the highest NAD-dependent IRED activity yet reported. These results highlight the ability of the artificial selection system to obtain variant enzymes where multiple kinetic parameters are enhanced simultaneously, resulting in enzymes with optimal activity towards the desired substrate. Furthermore, while identification of MsIRED_C_ by Borlinghaus and Nestl^36^ required multiple rounds of mutagenesis and screening, we obtained the superior NAD-dependent variant MsIRED_S_ in a single round.

### Chemically-directed evolution of enzyme variants with modified chemoselectivity and positional selectivity

**Fig. 5:**
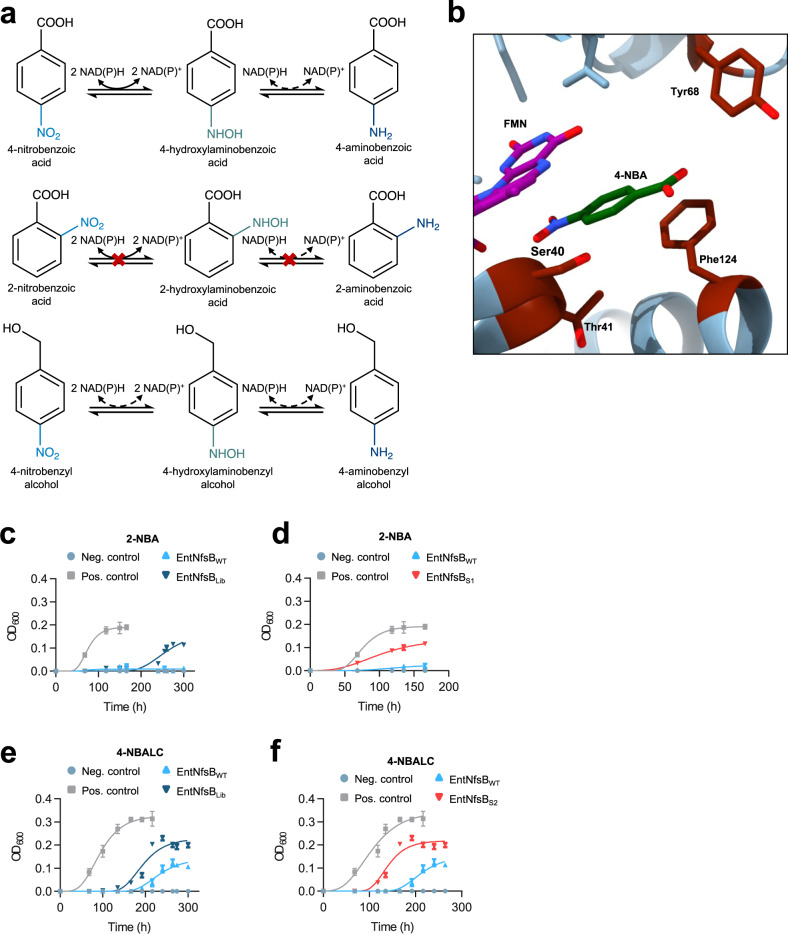
Chemically-directed selection of nitroreductase variants with altered substrate specificity. **a** Catalytic activities of EntNfsB (*Enterobacter cloacae* nitroreductase NfsB). EntNfsB is able to reduce several nitroaromatic compounds, such as 4-nitrobenzoic acid (4-NBA), using NADH or NADPH as cofactors. EntNfsB_WT_ is also able to reduce 4-nitrobenzyl alcohol (4-NBALC) less efficiently, but does not display any activity towards 2-nitrobenzoic acid (2-NBA). **b** Substrate binding site of EntNfsB_WT_ with 4-NBA bound (PDB code 5J8G). Residues 40, 41, 68, and 124 were close to the substrate, and did not contact the FMN group essential for catalysis. Anaerobic culture of AL cells transformed with the library EntNfsB_Lib_ (pLS169) in M9 glucose medium with 2-NBA (**c**) or 4-NBALC (**e**). Controls are as in Fig. 1. **d** Anaerobic culture of AL cells transformed with EntNfsB_S1_ (with substitutions S40A, T41I, and F124A; pLS169_1), the variant with activity towards 2-NBA, in M9 glucose medium supplemented with 2-NBA. **f** Anaerobic culture of AL cells transformed with EntNfsB_S2_ (with substitutions T41L, Y68L, and F124L; pLS169_3), the variant with improved activity towards 4-NBALC, in M9 glucose medium supplemented with 4-NBALC. Data points of growth curves represent mean values, with error bars showing standard deviation; *n* = 3 biologically independent cultures for all timepoints of growth curves. Source data are provided as a Source Data file.

We aimed to apply the artificial selection pressure to target the evolution of enzyme activity towards non-native chemicals. Nitroreductases can synthesize aromatic hydroxylamines or amines (pharmaceutical and agrichemical precursors) from low-cost and readily available nitroaromatics^37^, can activate nitroaromatic anticancer prodrugs^38,39^, and can be used for bioremediation of soils contaminated with explosives such as TNT^40,41^. However, these applications often require the tailoring of natural nitroreductases to improve their catalytic properties. We targeted the classic *Enterobacter cloacae* nitroreductase NfsB (EntNfsB), a type-I nitroreductase able to reduce several nitroaromatic compounds, including 4-nitrobenzoic acid (4-NBA), and which can accept NAD and NADP as cofactors^42^. We characterized the activity of the WT enzyme, EntNfsB_WT_, with 4-NBA, 4-nitrobenzyl alcohol (4-NBALC), and 2-nitrobenzoic acid (2-NBA), finding that it was able to reduce 4-NBA and 4-NBALC but not 2-NBA (Fig. 5a, Supplementary Fig. 6, and Supplementary Table 2). The activity towards 4-NBALC was, however, considerably lower than towards 4-NBA, which prevented the accurate determination of kinetic parameters.

We performed full combinatorial saturation mutagenesis of residues S40, T41, Y68, and F124 of EntNfsB (generating a library (EntNfsB_Lib_) of 16.8 million unique genetic variants), chosen based on their proximity to the substrate-binding pocket and their lack of direct contact with the essential FMN^43^ (Fig. 5b). AL cells were transformed with the library and cultured anaerobically in a minimal medium supplemented with 2-NBA or 4-NBALC (Fig. 5c, d). We identified a different variant from the cells grown in the presence of each of the substrates. The variant selected in the cultures with 2-NBA (EntNfsB_S1_) contained substitutions S40A, T41I, and F124A, while the variant selected in the cultures with 4-NBALC (EntNfsB_S2_) contained T41L, Y68L, and F124L substitutions. In order to determine the product generated by the EnfNfsB variants, the fermentation broth of AL cells transformed with plasmids expressing either EntNfsB_S1_ or EntNfsB_S2_ and grown anaerobically in minimal medium with 2-NBA or 4-NBALC (Fig. 5e, f) respectively was characterized by ^1^H-NMR (Supplementary Table 1). Interestingly, the observed product signals matched those of the aromatic amines corresponding to 2-NBA or 4-NBALC, despite a previous report indicating that EntNfsB_WT_ can only reduce 4-NBA to 4-hydroxylaminobenzoic acid, and not all the way to the amine 4-aminobenzoic acid^42^. That report only assayed the in vivo activity up to 24 h. It is possible that the highly reducing conditions inside AL cells, combined with longer periods of incubation under anaerobic conditions, allow for the formation of the amine products. Enzymatic assays confirmed that EntNfsB_S1_ was able to reduce 2-NBA (*K*_m_ = 0.808 mM, *k*_cat_ = 28.4 min^−1^ and *k*_cat_/*K*_m_ = 585 M^−1^ s^−1^), and that EntNfsB_S2_ could reduce 4-NBALC more efficiently than WT EntNfsB, allowing determination of kinetic parameters (*K*_m_ = 1.111 mM, *k*_cat_ = 205.0 min^−1^ and *k*_cat_/K_m_ = 3075 M^−1^ s^−1^, Supplementary Fig. 6, Supplementary Table 2). Both variants retained the ability to reduce 4-NBA. Therefore, EntNfsB_S1_ is active on both 2-NBA and 4-NBA, unlike any previously reported nitroreductase. The ability to engineer nitroreductase selectivity and promiscuity in this way could prove useful in applications including activation of anticancer prodrugs with multiple nitro groups, and bioremediation of soils contaminated with nitroaromatics, where a complex mixture of different compounds is often found, and therefore the ability to act on multiple isomers is desirable. The isolation of EntNfsB_S1_ and EntNfsB_S2_ demonstrates chemically-directed evolution as a powerful application of the artificial selection system, using an external supply of target substrates to direct both improvement of an existing poor activity, and acquisition of an activity with a non-native substrate.

### Coupling artificial selection to genetic design optimization yields the best-performing synthetic isopropanol pathway

We anticipated that the artificial selection system should be readily applicable to more complex systems than individual enzymes, such as metabolic pathways. Furthermore, the selection pressure should act not only on the sequences of enzymes, but in general on any genetically-encoded trait that can be linked to the generation of NAD^+^, such as regulatory sequences controlling gene expression. These sequences, particularly promoters and ribosome binding sites (RBSs), are important in the design and optimization of heterologous/synthetic metabolic pathways, as they control the amount of each enzyme that is produced. To maximize production, high yet balanced flux across the pathway is required, while avoiding the accumulation of intermediates^44,45^ and expression-associated metabolic burden^46^. Finding the best combination of regulatory elements for a specific pathway is not trivial, often requiring multiple rounds of laborious and time-consuming screening.

**Fig. 6:**
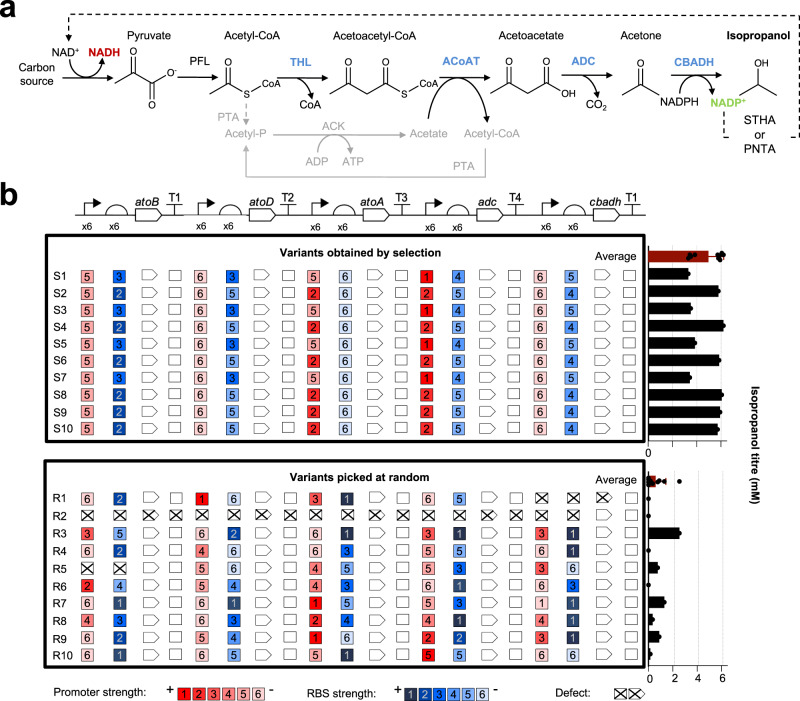
Selection of an isopropanol production pathway with optimized regulatory elements. **a** A synthetic isopropanol production pathway for *E. coli*. Thiolase (acetyl-CoA acetyltransferase) THL (encoded by *atoB*) catalyzes the condensation of two acetyl-CoA molecules to one acetoacetyl-CoA, releasing one free CoA molecule. Acetoacetyl-CoA transferase ACoAT (comprising two subunits encoded by *atoA* and *atoD*) transfers the CoA group from acetoacetyl-CoA to acetate, yielding acetoacetate and acetyl-CoA. Acetoacetate is decarboxylated by acetoacetate decarboxylase ADC (encoded by *adc*), resulting in acetone and CO2. Finally, acetone is reduced to isopropanol by CBADH. The acetate consumed by ACoAT can be regenerated from the resulting acetyl-CoA by phosphate acetyltransferase PTA and acetate kinase ACK. The NADP^+^ generated during the reduction of acetone can be employed by transhydrogenases STHA and PNTA to regenerate the NAD^+^ required to sustain anaerobic growth. **b** A library of isopropanol pathway-encoding plasmids was combinatorially assembled using mixtures of promoters and RBSs, expected to cause a diversity of performance in terms of flux, accumulation of intermediates, and expression-associated metabolic burden. 10 ‘S’ pathway variants were isolated by redox selection in *E. coli* AL cells and 10 ‘R’ pathway variants were isolated at random. Sequencing was used to determine the parts present in each case, which are shown aligned below the corresponding part in the design. Only two different permutations of parts were identified among the S variants, whereas all 10R variants were unique. Two R variants were defective, lacking one or more expected parts, whereas all S variants were complete. For comparison of isopropanol production, AL cells containing the S and R variants were cultured in M9 glucose medium for 17 h, then individual and average isopropanol titers in culture broths of both groups were determined. For both variants obtained by selection and variants picked at random, *n* = 10 biologically independent samples. In each set, black bars represent individual biological replicates, and the red bar represents the mean value with standard deviation indicated by the error bar obtained from the corresponding set of individual biological replicates. Source data are provided as a Source Data file.

Isopropanol is a widely used solvent, additive, and platform chemical with a large market, conventionally manufactured by petrochemical routes. We sought to develop an isopropanol production pathway and prototype whole-cell biocatalyst superior to the natural and engineered examples reported previously^47^ (Fig. 6a). A library (MP_Lib_) of variants of pathway-encoding plasmids, differing in the regulatory elements (promoters and RBSs) controlling each gene, was constructed by combinatorial DNA assembly using the Start-Stop Assembly^48^ system (Fig. 6b), giving a library of 60.4 million different variants. While only one enzyme in the pathway (CBADH) directly generates NADP^+^ (converted to NAD^+^ by transhydrogenases), the rate depends on the flux through the entire pathway, which in turn depends on a combination of regulatory genetic elements giving optimal expression of all enzymes, without causing problematic overexpression. This coupling allows the artificial selection pressure to be applied to the identification of optimal genetic designs.

AL cells were transformed with MP_Lib_ and grown anaerobically on minimal medium agar plates, with gluconate as a carbon source instead of glucose, because the maximum theoretical yield of isopropanol from gluconate is greater. Colonies of transformed AL cells were visible after 65 h of anaerobic incubation. We verified the ability of 10 colonies to grow anaerobically after inoculating them into minimal medium liquid cultures, and confirmed the presence of isopropanol in the fermentation broth by ^1^H-NMR (Supplementary Table 1).

We compared the combinations of regulatory elements and the isopropanol titer of 10 ‘S’ variants obtained by artificial selection with those of 10 ‘R’ variants picked from the library at random without artificial selection. We found only two different combinations of regulatory elements among the S variants (MP_S1_ and MP_S2_), whereas the 10R variants were all different, and included defective variants lacking one or more of the genes, which can arise during DNA assembly (Fig. 6b). In these minimal culture conditions, the S variants gave an average isopropanol titer of 4.97 mM after 17 h of incubation, which was significantly greater (*t*(9) = 9.22 and *p* = 1.54 × 10^−8^, or excluding defective variants *t*(9) = 6.82 and *p* = 4.19 × 10^−6^) than the average of 0.60 mM produced by the R variants (Fig. 6b). This indicates that the artificial selection pressure had acted both to eliminate defective variants and to favour specific combinations of genetic elements leading to maximized production of isopropanol.

We compared the performance of the best isopropanol pathway variant MP_S1_ with the best isopropanol pathway reported previously, by Hanai and coworkers^47^, using the same growth medium and culture conditions. The previous best 43.5% of maximum theoretical isopropanol yield during the production phase (with 45 mM titre) was surpassed here by WT *E. coli* cells transformed with MP_S1_, which achieved 56% at the same point (with 62 mM titre), the highest isopropanol yield reported so far for any organism, including both native producers and engineered strains.

## Discussion

Known natural enzymes and other proteins encompass extremely diverse catalysts, structures, materials, and molecular machines, yet necessarily these represent a tiny subset of all possible proteins. Automated chemical synthesis can readily provide DNA encoding arbitrary protein sequences, so identifying novel functionalities in protein sequence space is effectively solely a search problem. Searches of such astronomically large and highly multidimensional design spaces cannot be exhaustive, so success is a function of start points, search strategies, and throughput; as well as the structure of the space.

Natural evolution and laboratory evolution alike use existing proteins as start points, and generate new functional proteins by modest numbers of modifications. Initiating searches for new proteins from these proximal start points greatly decreases the scale of the search, increasing feasibility. However, it also means that ‘new’ proteins are very similar to their parents. In this context, new functional proteins identified from metagenomic resources^49^ also represent additional start points to access new regions of sequence space. It is not currently possible to make large jumps in sequence space to islands of functionality distant from the natural repertoire, a limitation that might be overcome by the future development of *de novo* enzyme design, allowing exotic and truly novel proteins to be identified. In this direction, artificial intelligence (AI) and machine learning (ML) are already being applied to directed evolution and protein engineering^50,51^. Encouraging progress towards solving the 50-year-old protein folding problem has recently been made using AI^52^, although predicting function is much more difficult. When developing new or improved enzyme activities, there is currently no substitute for experimental evaluation, so throughput is key.

Isolation and screening of variants using robotic automation is the current state of the art in protein engineering, but remains much slower than library generation, so is usually the bottleneck. Technologies like FACS and microfluidics achieve higher throughputs, but are less widely applicable. Artificial selection provides the highest throughput, because variants do not need to be physically separated for individual evaluation, and in some cases because beneficial mutations can continuously accumulate without intervention. Selection is part of classical strain improvement^53^, a longstanding approach re-popularized in recent years, with the benefit of modern genome sequencing, as Adaptive Laboratory Evolution (ALE)^54^. Over time, artificial selections^6^ have enabled powerful technologies, with limited scopes, including Systematic Evolution of Ligands by Exponential Enrichment (SELEX)^55,56^ for generation of high-affinity binding aptamers, Compartmentalized Self-Replication (CSR)^57^ for directed evolution of DNA polymerases, and Phage-Assisted Continuous Evolution (PACE)^58^, which supports selection of factors linked to gene expression.

Previous studies have shown that *E. coli* cells with defective anaerobic fermentation can be used to select redox-active enzymes or pathways acting on substrates connected to native metabolism^8–11,13^. Here, we have demonstrated that such a metabolic defect can be exploited as an unusually widely applicable selection system acting on metabolically isolated enzymes by providing an external electron acceptor in the culture medium. The outcome of the selection can be directly controlled by simply replacing the externally provided substrate, enabling an easily-tunable, chemically-driven selection system. The selection pressure acts directly on NAD^+^ regeneration, so is directly applicable to NAD-dependent oxidoreductases, a very large class of enzymes^59^. The diverse viable growth rates observed among selected variants and controls, and diverse kinetic parameters among selected variants, indicate that the selection has a wide dynamic range. Furthermore, the selection is highly amenable to extension through coupling. This first study includes optional coupling to NADP-dependent activities via transhydrogenases; coupling to non-redox enzymes (in the isopropanol pathway here, but many pathways and cascades contain at least one NAD(P)H-dependent step); and coupling to genetic design, illustrating the broad scope of selection, not limited to protein sequences. These results demonstrate that the system will be applicable to diverse problems in industrial biotechnology, and especially in biocatalysis.

Direct applications of examples described here include the development of alcohol dehydrogenases (ketoreductases) for production of chiral alcohols^60^, imine reductases (especially the subset with reductive aminase activity) for production of chiral amines^61^, and development of isopropanol-producing microbial strains. The artificial selection could be applied widely in the fields represented by these specific examples; the development of enzymes and cascades for the synthesis of pharmaceutical and agrichemical precursors, and whole-cell biocatalysts required for sustainable biomanufacture of commodity chemicals. Besides laboratory development, nature is still a very important source of biocatalysts^49^. Identifying new enzymes from metagenomic sources is conceptually similar to identifying desired variants from synthetic libraries, and the selection is well suited to both.

The great throughput provided by artificial selection is more than a ‘brute force’ alternative to computational design: The libraries in this study are too large to screen by conventional methods, but cutting library sizes requires assumptions. For example, the CBADH and TBADH variants which outcompeted all others (and previous reports) under selection pressure were not part of the much smaller computationally-designed libraries. Furthermore, the capacity to include an additional residue in the initial design obviated subsequent recovery of activity.

Even the throughput provided by artificial selection is not a panacea given the size of protein sequence space. It will be very powerful to combine selection with AI/ML approaches, iteratively using AI/ML for library design and artificial selection to rapidly search libraries, and hence rapidly progress through design space. Artificial selection could be coupled to an in vivo mutagenesis system, such as OrthoRep^62^ or eMutaT7^63^, to achieve fully automated and continuous in vivo evolution. In time, these approaches will support the transition from the fossil economy to the sustainable, circular bioeconomy, inform protein and enzyme design principles, and ultimately enable the development of *de novo* design of enzymes and other systems.

## Methods

### Construction of *E. coli* mutant strains

*E. coli* strain JW1228, the Δ*adhE* mutant from the Keio collection^64^, was used as the starting point for construction of multiple strains. The pMAK705 allele exchange method^65^ was used to construct the AL (Δ*adhE* Δ*ldhA*), ALP (Δ*adhE* Δ*ldhA* Δ*pntA*), ALS (Δ*adhE* Δ*ldhA* Δ*sthA*) and ALPS (Δ*adhE* Δ*ldhA* Δ*pntA* Δ*sthA*) strains; using pMAK705^65^-derived plasmids targeting *ldhA* (pLS63), *pntA* (pLS40) and *sthA* (pLS39) (Supplementary Table 3). Briefly, cells were transformed by heat shock at 37 °C, plated onto LB agar plates with chloramphenicol, and incubated at 30 °C. Single colonies were used to inoculate LB medium with chloramphenicol, and incubated at 30 °C overnight. Serial dilutions prepared from the overnight cultures were plated onto LB agar plates with chloramphenicol and incubated at 44 °C (the non-permissive temperature for replication of these plasmids) with chloramphenicol selection to isolate single crossover (cointegrate) clones, then subcultured and incubated at 30 °C (the permissive temperature) without selection to allow cointegrate resolution and to allow the arising double crossover clones to grow, and finally subcultured and incubated at 44 °C without selection to eliminate the plasmid. Mutants were verified by antibiotic resistance profile and PCR screening of the target locus. Firstly, pLS63 was used to derive the AL strain from JW1228. The AL strain was then subjected to the same protocol with pLS40 or pLS39, yielding strains ALP and ALS, respectively. Finally, ALS was treated with pLS40 to obtain the quadruple mutant strain ALPS.

### Construction of plasmids

Plasmids are listed in Supplementary Table 3. Oligonucleotides are listed in Supplementary Table 4. *E. coli* DH5α was used as a cloning host. Restriction digestion and ligation reactions were performed as recommended by the manufacturer (New England Biolabs). All constructed plasmids were isolated and verified by sequencing.

Sequences encoding ADHE (AIN31697.1 [https://www.ncbi.nlm.nih.gov/protein/AIN31697.1]) and BDHA (CAB12443.1 [https://www.ncbi.nlm.nih.gov/protein/CAB12443.1]) were PCR-amplified from genomic DNA of *E. coli* BW25113 and *Bacillus subtilis* 168, respectively. Sequences encoding CBADH (AAA23199.2 [https://www.ncbi.nlm.nih.gov/protein/AAA23199.2/]), BUDC (AAC78679.1 [https://www.ncbi.nlm.nih.gov/protein/AAC78679.1]), TADH (ACD50896.1 [https://www.ncbi.nlm.nih.gov/protein/ACD50896.1]), TBADH (CAA46053.1 [https://www.ncbi.nlm.nih.gov/protein/CAA46053.1]), MsIRED (AGC43099.1 [https://www.ncbi.nlm.nih.gov/protein/AGC43099.1]) and EntNfsB (AAA62801.1 [https://www.ncbi.nlm.nih.gov/protein/AAA62801.1]) were codon-adapted suitably for expression in *E. coli* and chemically synthesized (IDT or DNA2.0) (Supplementary Table 5). Plasmids for expression of these enzymes were constructed by cloning the coding sequences into pUC19. Synthetic sequences encoding ADHE, BDHA, CBADH, BUDC, TADH, TBADH, and MsIRED were PCR amplified, the PCR products were digested with SphI and BamHI, and the appropriate fragments were purified and ligated with pUC19 vector (previously linearized by treatment with SphI and BamHI) using T4 DNA ligase. For EntNfsB, the pUC19 vector backbone was PCR amplified with primers introducing BbsI restriction sites. The pUC19 PCR product was mixed with the synthetic sequence encoding EntNfsB and assembled in a Golden Gate reaction in the presence of DpnI. Golden Gate reactions were performed in a total reaction volume of 20 μL in 1 × T4 DNA ligase buffer, comprising 50 fmol of backbone (linearized pUC19 with BbsI restriction sites obtained as described above), 50 fmol of the DNA fragment to be cloned, 1 U/μL of BbsI-HF restriction enzyme and 20 U/μL of T4 DNA ligase. The reaction mixture was then incubated in a thermocycler with 30 cycles alternating 5 min steps at 37 °C with 5 min steps at 16 °C, before performing a single final deactivation step at 65 °C for 20 min. pLS131, encoding the previously-reported^36^ MsIRED_C_ variant, was generated by inverse PCR using pLS130 (encoding WT MsIRED) as template and primers designed to introduce the appropriate substitutions (N32E, R33Y, T34E, K37R, L67I, and T71V) followed by self-ligation of the PCR product using T4 DNA ligase. To construct plasmids for expression of hexahistidine-tagged versions of MsIRED, EntNfsB, and their variants, the coding sequences were PCR-amplified using primers containing a C-terminal hexahistidine tag and BbsI restriction sites (Supplementary Table 5). The resulting PCR products were mixed with pUC19 vector backbone previously PCR-amplified with primers introducing BbsI restriction sites and used to perform a Golden Gate assembly reaction as described above. To construct plasmids for expression of hexahistidine-tagged versions of CBADH, TBADH, and their variants, coding sequences were amplified by PCR using primers designed to introduce NdeI and BlpI restriction sites. PCR products and the pET28 vector were digested using NdeI and BlpI, and the appropriate purified fragments were ligated using T4 DNA ligase, resulting in pET28-derived plasmids encoding enzymes with N-terminal hexahistidine tags.

To construct allele exchange directed mutagenesis plasmids derived from pMAK705, two 500 bp sequences, one upstream and one downstream of the target gene, were PCR-amplified from genomic DNA of *E. coli* BW25113. The resulting PCR products were fused using overlap extension PCR using primers incorporating BamHI and HindIII sites. The product of the overlap extension PCR was digested with BamHI and HindIII, and ligated with linearized pMAK705 (obtained by treating pMAK705 with BamHI and HindIII) using T4 DNA ligase.

### Prediction of residues involved in nicotinamide cofactor preference

CSR-SALAD^25^ with standard parameters was used to predict residues important in determining the nicotinamide cofactor preference of CBADH, TBADH, and MsIRED. The inputs were the available crystal structures for CBADH and TBADH (PDB codes 1KEV [10.2210/pdb1KEV/pdb] and 1YKF [10.2210/pdb1YKF/pdb], respectively), or a homology model of MsIRED generated with SWISS-MODEL^66^ using the crystal structure of *Streptomyces kanamyceticus* imine reductase as starting model (PDB code 3ZHB [10.2210/pdb3ZHB/pdb]).

### Construction of full combinatorial saturation mutagenesis libraries

Libraries of variants of CBADH, TBADH, MsIRED, and EntNfsB were generated by full combinatorial saturation mutagenesis of the targeted positions using either inverse PCR (for CBADH and TBADH) or Golden Gate assembly (for MsIRED and EntNfsB). For inverse PCR reactions, a pair of primers were designed for each reaction, with degenerate N mixtures of nucleotides at each position targeted for saturation mutagenesis. The 5′ end of one primer in each pair was phosphorylated (Supplementary Table 4). The reactions were carried out with Phusion High-Fidelity DNA polymerase (New England Biolabs) using as template the pUC19-derived expression plasmid for each parental coding sequence, described above. The reactions were performed according to the manufacturer’s instructions in a total volume of 50 μL. PCR products were treated with DpnI, purified, and circularized by self-ligation with T4 DNA ligase. For Golden Gate assembly reactions^67^, first the pUC19 vector backbone was amplified by PCR with primers with overhangs designed to incorporate matching BbsI restriction sites. The parent coding sequences were amplified with one or more pairs of primers containing degenerate N mixtures of nucleotides at the positions targeted for saturation mutagenesis and overhangs matching the corresponding BbsI restriction sites. The resulting PCR amplified fragments contained the targeted positions and covered the entire coding sequence, such that assembly would lead to libraries of full-length coding sequences. The amplified vector backbone and PCR fragments were used to carry out a Golden Gate assembly reaction in the presence of DpnI as previously described. Successful genetic diversification was verified by sequencing 10 random isolated clones of each library, obtaining in all cases 10 different sequences (Supplementary Tables 6−9).

### Construction of the library of isopropanol production pathways

The *atoB*, *atoD*, and *atoA* coding sequences were obtained by PCR amplification from *E. coli* strain BW25113 genomic DNA. The *adc* coding sequence was obtained by PCR amplification from *Clostridium acetobutylicum* ATCC 824 genomic DNA. The sequence encoding CBADH (*cbadh*) was obtained by PCR amplification from the pLS6 plasmid. In all cases, primers designed to introduce BsaI overhangs were used.

Individual parts, expression units, and full metabolic pathways were constructed by hierarchical Start-Stop Assembly^48^. Firstly, functional genetic parts were cloned in the Level 0 storage plasmid pStA0 by mixing the PCR amplified fragments with BsaI-digested vector and ligating with T4 DNA ligase. Level 0 mixtures of six different promoters and 6 different RBSs (described previously^48^) were prepared as equimolar mixtures of the plasmids containing these parts. Libraries of Level 1 plasmids (each containing whole expression units comprising a promoter, an RBS, a functional gene, and a transcriptional terminator) were obtained by Start-Stop Assembly reactions. For each functional gene, the reaction was performed in a total reaction volume of 20 μL in 1 × T4 DNA ligase buffer, and included 20 fmol of the destination vector (pStA1AB, pStA1BC, pStA1CD, pStA1DE or pStA1EZ), 40 fmol of the mixture of Level 0 plasmids carrying promoters, 40 fmol of the mixture of Level 0 plasmids carrying RBSs, 40 fmol of Level 0 plasmid carrying the corresponding gene, 40 fmol of Level 0 plasmid carrying a terminator out of four different possibilities (as shown in Supplementary Table 3), T4 DNA ligase buffer, 20 U/μL of T4 DNA ligase and 0.5 U/μL of SapI. The reaction mixture was incubated using a thermocycler for 30 cycles of 37 °C for 5 min and 16 °C for 5 min, before a single final step of deactivation at 65 °C for 20 min. The resulting products were used to transform *E. coli* DH5α cells, and plasmid DNA was isolated. The Level 2 library (containing variants of the full metabolic pathway differing in the combination of promoters and RBSs) was generated with similar Start-Stop Assembly reactions, but the BsaI restriction enzyme was used instead of SapI.

### Anaerobic cultures

*E. coli* AL, ALS, ALP, or ALPS cells were transformed with individual plasmids encoding WT enzymes or variants, or plasmid libraries encoding variants of enzymes or variants of pathways. Transformed cells were transferred into 15 mL of M9 medium and incubated aerobically at 30 °C for 6 h to recover. Recovered transformant cells were then used at a 1:100 dilution to inoculate anaerobic M9 media containing 18 mM glucose or (where stated) gluconate, 100 μg/mL ampicillin or 50 μg/mL kanamycin as appropriate, 1 mM IPTG, and an external substrate as appropriate (Supplementary Table 10). Anaerobic cultures were incubated in a Don Whitley Scientific A35 anaerobic workstation containing anaerobic growth gas mix (10% CO_2_, 10% H_2_, and 80% N_2_). The culture medium was made anaerobic by placing it in the anaerobic workstation for 24 h before inoculation. Growth was monitored by measuring OD_600_. In all growth complementation experiments, cells transformed with pLS1 encoding *E. coli* ADHE were used as the positive control, and cells transformed with empty vector pUC19 were used as the negative control.

For artificial selection experiments performed with enzyme variant libraries, after the plateau phase of growth was reached, cells were subcultured under the same selective conditions to select variants supporting growth, and to dilute out other variants. Selected variants were isolated by culturing samples of the grown cells on LB agar plates containing the appropriate antibiotic and picking single colonies, which were sequenced to identify the variant.

For artificial selection on the solid medium of the library of variants of the isopropanol pathway MP_Lib_, AL cells transformed with the library were plated onto anaerobic M9 minimal medium agar plates supplemented with 18 mM gluconate, 50 μg/mL kanamycin, and 1 mM IPTG. The plates were made anaerobic by placing them in the anaerobic workstation for 24 h before plating cells.

Growth curves were generated using GraphPad PRISM by fitting a Gompertz growth equation to the OD_600_ values measured at different time points.

### Evaluation of isopropanol pathway variants

*E. coli* AL cells were transformed with MP_Lib_ and plated on both aerobic and anaerobic M9 minimal medium agar plates supplemented with 18 mM gluconate, 50 μg/mL kanamycin, and 1 mM IPTG. The redox selection pressure acts under anaerobic conditions. Plasmid DNA was isolated from 10 colonies from the aerobic plates (random ‘R’ variants) and 10 colonies from the anaerobic plates (selected ‘S’ variants). Purified plasmids were sequenced and used to retransform *E. coli* AL cells. To compare isopropanol production among these clones (Fig. 6), transformed cells were used to inoculate M9 minimal medium supplemented with 18 mM gluconate, 50 μg/mL kanamycin, and 1 mM IPTG and incubated aerobically for 17 h at 37 °C and 250 rpm shaking, after which cultures were centrifuged and the supernatant was analyzed by ^1^H-NMR. The isopropanol titers were compared by performing a one-tailed t-test, and a large effect size was confirmed by calculating Cohen’s D (*D* = 4.123, or excluding defective variants *D* = 3.519). Statistical tests were performed in R.

In order to compare the isopropanol production of MP_S1_ (the best isopropanol pathway variant obtained by artificial selection in this study) with a previous report^47^, the culture conditions used in that previous report were replicated. *E. coli* BW25113 cells were transformed with pLS60_1, encoding pathway variant MP_S1_. Transformed cells were plated onto LB agar plates supplemented with 50 μg/mL kanamycin and incubated aerobically overnight at 37 °C. Individual colonies were picked and used to inoculate precultures of 5 mL SD-7 medium^47^ with 111 mM glucose. Cells were cultured aerobically for 17 h at 37 °C and 250 rpm shaking. Then, 250 μL of the preculture was used to inoculate 25 mL of SD-8 medium^47^ with 111 mM glucose in 250 mL flasks with baffles. After culturing cells aerobically for 9 h (because the previous study^47^ reported yield at this point, due to it being the most productive phase of the culture) at 37 °C and 250 rpm shaking, the isopropanol titer in the culture broth was determined by ^1^H-NMR. All cultures were supplemented with 50 μg/mL kanamycin.

### Analysis of culture broths by ^1^H-NMR

Samples of cultures were centrifuged for 15 min at 8000 × g to pellet the cells. 0.5 mL of each supernatant was removed and mixed with 0.5 mL of NMR buffer (75 mM Na_2_HPO_4_ buffer at pH 7.4, 4.6 mM 3-(trimethylsilyl)-[2,2,3,3-2H4]-propionate (TSP) and 20% (v/v) D_2_O). The resulting solution was transferred into a 3 mm NMR tube. 1D ^1^H-NMR spectra were acquired with TopSpin 3 on a Bruker Avance III 400 MHz spectrometer operating at 293 K using the zg30 pulse sequence. A total of 16 scans for each sample were collected into 32,768 data points with a spectral window of 20 ppm. Additionally, for each metabolite of interest, spectra of M9 medium supplemented with 10 mM of the metabolite were acquired in order to determine characteristic peaks of each metabolite (Supplementary Table 11 and Supplementary Fig. 7). The spectra were processed with the MestReNova suite. After performing phase and baseline correction, spectra were calibrated to the TSP signal at δ 0 ppm. Metabolite assignment was confirmed with spiking experiments. The concentration of each metabolite of interest was calculated by integrating the characteristic signal of each compound and comparing it to the area of the signal corresponding to TSP, taking into account the different number of protons corresponding to each signal. For each analysis, metabolite concentrations were measured in each of three independent replicate cultures, and the mean of these values was determined.

### Nickel-affinity chromatography purification of hexahistidine-tagged enzymes

*E. coli* BL21(DE3) cells were transformed with pET28a or pUC19 expression plasmids encoding hexahistidine-tagged proteins, allowed to recover at 37 °C for 1 h, then plated on LB agar plates supplemented with 50 μg/mL kanamycin or 100 μg/mL ampicillin and incubated at 37 °C to select transformants. Individual colonies were inoculated in 10 mL of LB broth with 50 μg/mL kanamycin or 100 μg/mL ampicillin and cultured overnight at 37 °C and 230 rpm. Cells were then inoculated at a 1:1000 ratio in LB with 50 μg/mL kanamycin or 100 μg/mL ampicillin and incubated at 37 °C and 230 rpm until an OD_600_ of 0.6 was reached. Then, protein expression was induced with 400 μM IPTG and incubated overnight at 18 °C and 230 rpm. Cells were harvested by centrifugation at 4000 × g for 30 min at 4 °C and resuspended in 50 mL of purification buffer (50 mM Tris-HCl at pH 7.4, 500 mM NaCl, 10% (w/v) glycerol and 1 mM TCEP) supplemented with Base Muncher DNAse, lysozyme, one cOmplete Protease Inhibitor Tablet per liter of culture and, in the case of nitroreductases, 100 μM flavin mononucleotide (FMN). After lysing cells by ultrasonication, the lysate was centrifuged at 21,000 × g for 1 h at 4 °C to pellet cell debris. The supernatant was filtered through a 0.21 μm filter and loaded onto a 5 mL HisTrap column (GE HealthCare). The column was extensively washed with purification buffer and eluted with a gradient of 25–500 mM imidazole. Eluted fractions were tested for enzymatic activity, and those containing the activity of interest were pooled and subjected to gel filtration in a Superdex 200 column (GE HealthCare) in buffer containing 50 mM Tris-HCl at pH 7.4 and 150 mM NaCl. The purity of peak fractions was assessed by means of SDS-PAGE. Additionally, for nitroreductase enzymes the presence of pure FMN-bound holoprotein was determined based on a A280/A454 ratio lower than 5. Purified proteins were concentrated to 40–80 μM, flash frozen with liquid nitrogen, and stored at −80 °C. All purification steps were carried out with an ÄKTA pure chromatography system (GE HealthCare).

### Enzymatic activity assays

Enzymatic assays at different substrate concentrations were performed at 37 °C with purified protein by monitoring NAD(P)H absorbance at 340 nm or 370 nm with an Eppendorf BioSpectrometer kinetic instrument. Assays were performed anaerobically for EntNfsB and its variants and aerobically for all other enzymes. All reactions were performed in a total volume of 200 μL for aerobic reactions and 400 μL for anaerobic reactions, with protein concentration ranging between 26 nM and 1.3 μM and saturating concentrations of cofactor (Supplementary Table 12). Oxidation reactions were performed in assay buffer A (50 mM Tris-HCl at pH 8.8 and 50 mM NaCl), while reduction reactions were carried out in assay buffer B (50 mM Tris-HCl at pH 7.4 and 50 mM NaCl). Reaction rates were determined using the initial linear rates. These were calculated from the change in absorbance using the known molar absorption coefficients^42^ of NADH and NADPH at 340 nm of ε_340_ = 6.22 × 10^3^ M^−1^ cm^−1^ and of NADH at 370 nm of ε_370_ = 2.66 × 10^3^ M^−1^ cm^−1^. Triplicate measurements were acquired for all initial reaction rates. For all reactions except those catalyzed by EntNfsB and its variants, equimolar amounts of cofactor and substrate are consumed as the reaction progresses, and thus the consumption of cofactor was directly converted to consumption of substrate. For reactions catalyzed by EntNfsB and its variants, the substrates were assumed to be reduced only to the corresponding hydroxylamines, due to previous reports indicating that the WT enzyme does not catalyze the reduction to amines in vitro^42^. Additionally, the reduction from the nitroso intermediate to the corresponding hydroxylamine was assumed to happen immediately. As a consequence, and considering that both the reduction to a nitroso compound and to a hydroxylamine require 2 NADH, calculations were performed on the basis of 4 mol of cofactor being consumed for each consumed mol of the substrate. Kinetic parameters were obtained by fitting a Michaelis-Menten model with GraphPad Prism, including substrate inhibition in the model when a reduction of initial rates was observed at higher substrate concentrations.

### Crystallization and structure solution

Purified CBADH_S_ and TBADH_S1_ were concentrated to ~10 mg/mL for crystallization with sitting drop vapour diffusion at 17 °C using commercial sparse matrix screens with a Mosquito robot (TTPLabTech). Crystallization hits were detected with a light-UV microscope and further optimized in manually set up hanging drop vapour diffusion experiments. Final CBADH_S_ crystals came from 900 mM sodium citrate, 100 mM imidazole at pH 8 mixed with an equal volume of protein solution. Final TBADH_S1_ crystals came from 20% (w/v) PEG 3 K and 100 mM sodium citrate at pH 5.5 mixed with an equal volume of protein solution. Crystals were soaked in the corresponding mother liquor with 1 mM NAD^+^, then cryoprotected for several minutes in mother liquor with 30% volume PEG 400 and flash-cooled in liquid nitrogen. X-ray diffraction data were collected at the Diamond Light Source synchrotron (Didcot, UK), and processed with the xia2 pipeline^68^ using AutoPROC^69^. Structures were solved by molecular replacement in Phaser^70^ using PDB 1KEV [10.2210/pdb1KEV/pdb]^71^ as a model for CBADH_S_ and PDB 1YKF [10.2210/pdb1YKF/pdb]^29^ for TBADH_S1_. The models were rebuilt in Coot^72^ with cycles of refinement in phenix.refine^73^, and validated with MolProbity^74^. Data collection and refinement information are presented in Supplementary Table 13.

### Reporting summary

Further information on research design is available in the Nature Research Reporting Summary linked to this article.

## Supplementary information

Supplementary Information

Reporting Summary

## Data Availability

The coordinates and structure factors for CBADH_S_ and TBADH_S1_ generated in this study have been deposited in the PDB under accession codes 6SCH (CBADH_S_) and 6SDM (TBADH_S1_). The DNA sequences of enzyme variants generated in this study have been deposited in GenBank under accession codes MW431031−MW431041. The CBADH_WT_, TBADH_WT_, *Streptomyces kanamyceticus* imine reductase and EntNfsB_WT_ structures used in this study are available in the PDB under accession codes 1KEV (CBADH_WT_), 1YKF (TBADH_WT_), 3ZHB (*Streptomyces kanamyceticus* imine reductase) and 5J8G (EntNfsB_WT_). Source data are provided with this paper.

## Source data

Source Data
