# Supplementary material for: Versatile selective evolutionary pressure using synthetic defect in universal metabolism
Source: Nat Commun. 2021 Nov 25;12:6859. doi: 10.1038/s41467-021-27266-9 (PMC8616928; doi:10.1038/s41467-021-27266-9)
Supplement: Supplementary file 1 — Supplementary Information [file 41467_2021_27266_MOESM1_ESM.pdf]

# Supplementary Information

## Versatile selective evolutionary pressure using synthetic defect in universal metabolism

Lara Sellés Vidal[1,2], James W. Murray[2] and John T. Heap*[1,2,3]

*Corresponding author: John T. Heap, email: john.heap@nottingham.ac.uk
[1] Imperial College Centre for Synthetic Biology, Imperial College London, London, SW7 2AZ, United Kingdom
[2] Department of Life Sciences, Imperial College London, London, SW7 2AZ, United Kingdom
[3] School of Life Sciences, The University of Nottingham, Biodiscovery Institute, University Park, Nottingham, NG7 2RD, United Kingdom

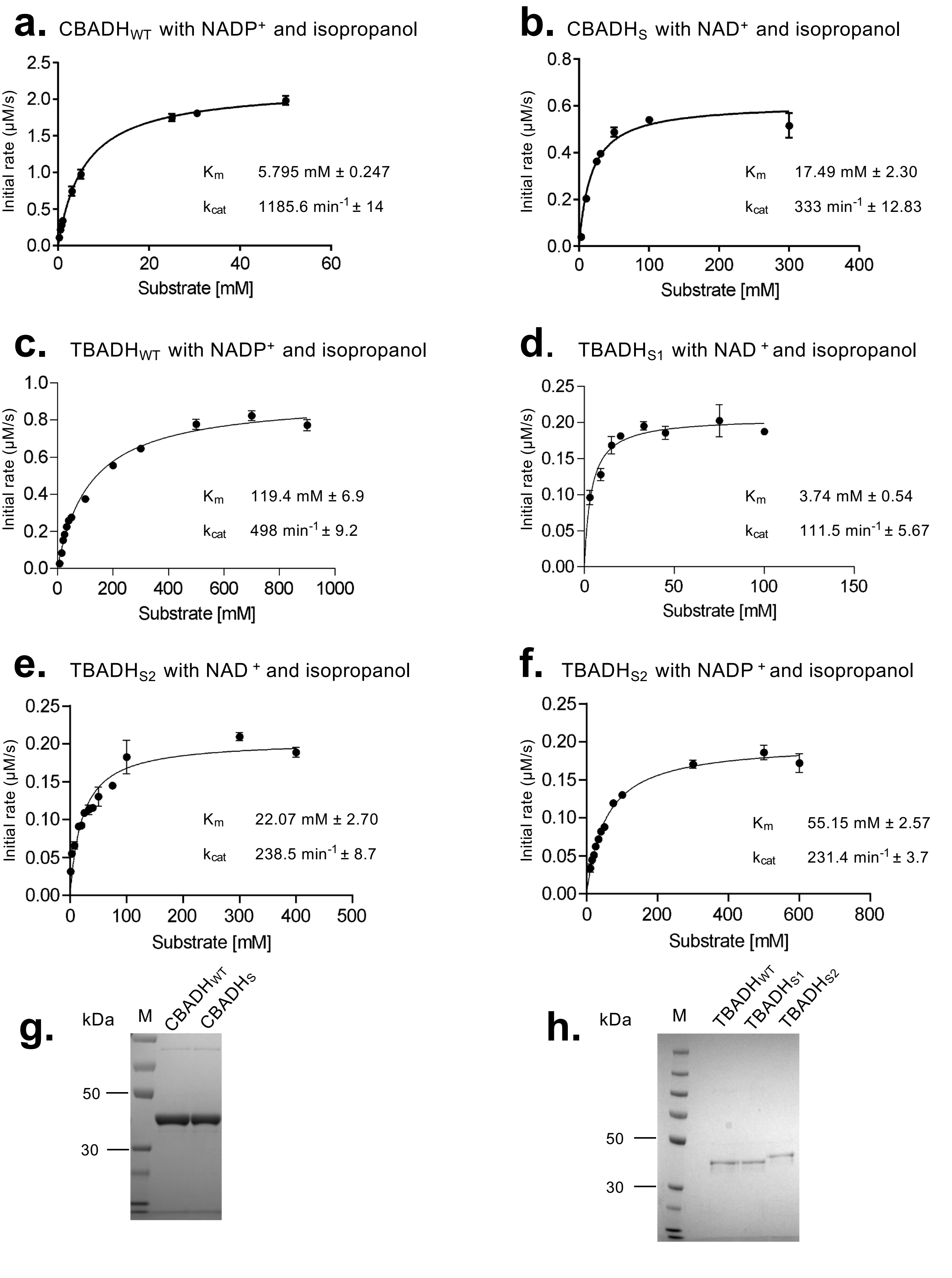


**Supplementary Figure 1. Enzymatic activity assays for CBADH and TBADH variants. a-f**, Initial reaction rates obtained for each enzyme by varying the isopropanol concentration at a fixed concentration of NAD$^+$ or NADP$^+$. A Michaelis-Menten model was fitted in all cases. Data points represent mean values, with error bars showing standard deviation. n=3 biologically independent assays for all substrate concentrations with both enzymes. Source data are provided as a Source Data file. **g, h**, SDS-PAGE confirming the purity of CBADH$_{WT}$, CBADH$_S$, TBADH$_{WT}$, TBADH$_{S1}$ and TBADH$_{S2}$ used for enzymatic assays. Active, pure proteins were successfully obtained with the described procedure from three independent preparations.

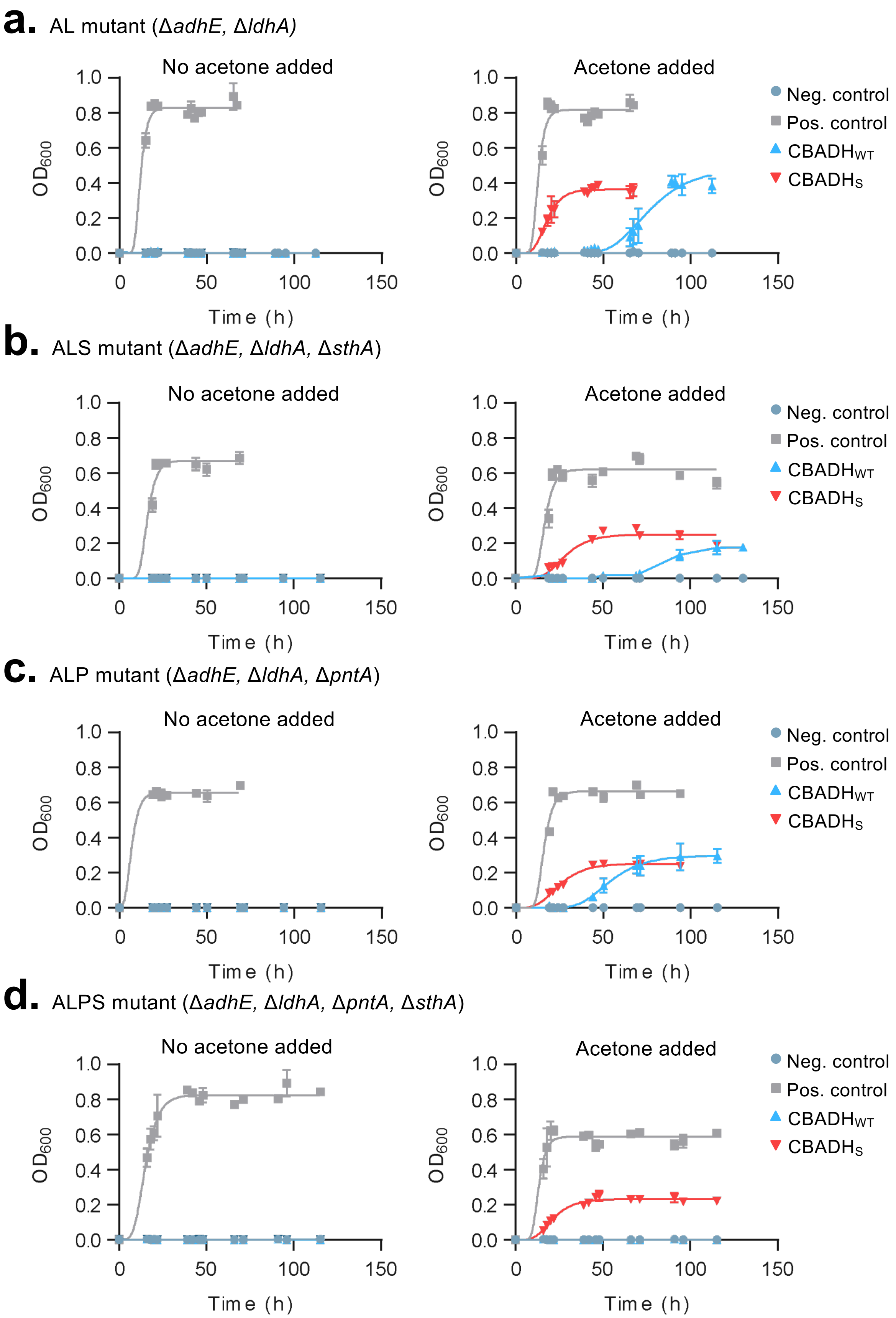


**Supplementary Figure 2. Anaerobic cultures of AL, ALS, ALP and ALPS cells containing various NAD and NADP-dependent oxidoreductases.** For each *E. coli* strain, anaerobic growth with (right panels) and without (left panels) acetone supplemented to the culture media was followed. **a.** AL mutant (Δ*adhE* Δ*ldhA*). **b.** ALS mutant (Δ*adhE* Δ*ldhA* Δ*sthA*). **c.** ALP mutant (Δ*adhE* Δ*ldhA* Δ*pntA*). **d.** ALPS mutant (Δ*adhE* Δ*ldhA* Δ*pntA* Δ*sthA*). Anaerobic growth of cells with at least one active transhydrogenase was recovered upon transformation of either an NAD or an NADP-dependent oxidoreductase. However, in the case of ALPS cells, where both transhydrogenase genes were deleted, only the NAD-dependent enzyme restored anaerobic growth, indicating that metabolic complementation by NADP-dependent enzymes is mediated by transhydrogenases. Data points of growth curves represent mean values, with error bars showing standard deviation; n=3 biologically independent cultures for all timepoints of growth curves. Source data are provided as a Source Data file.

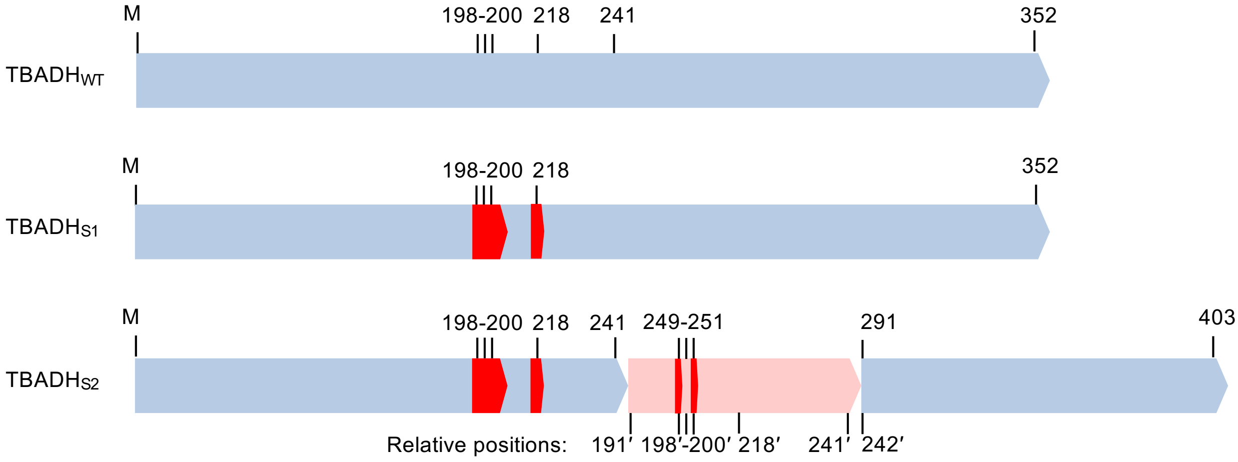


**Supplementary Figure 3. Overview of mutations in $TBADH_{S1}$ and $TBADH_{S2}$.** Absolute positions are shown above each sequence. Point mutations are shown in red. $TBADH_{S1}$ contains substitutions at all positions targeted for saturation mutagenesis (198, 199, 200 and 218). $TBADH_{S2}$ contains substitutions at all targeted positions and a duplication (shown in pink) of residues 191-241 inserted between positions 241 and 242 of the original sequence. Positions relative to $TBADH_{WT}$ are shown below $TBADH_{S2}$ with a prime symbol. The insertion contains a second copy of the targeted positions, two of which were also substituted. These were positions 249 and 251, corresponding to positions 198’ and 200’ of $TBADH_{WT}$.

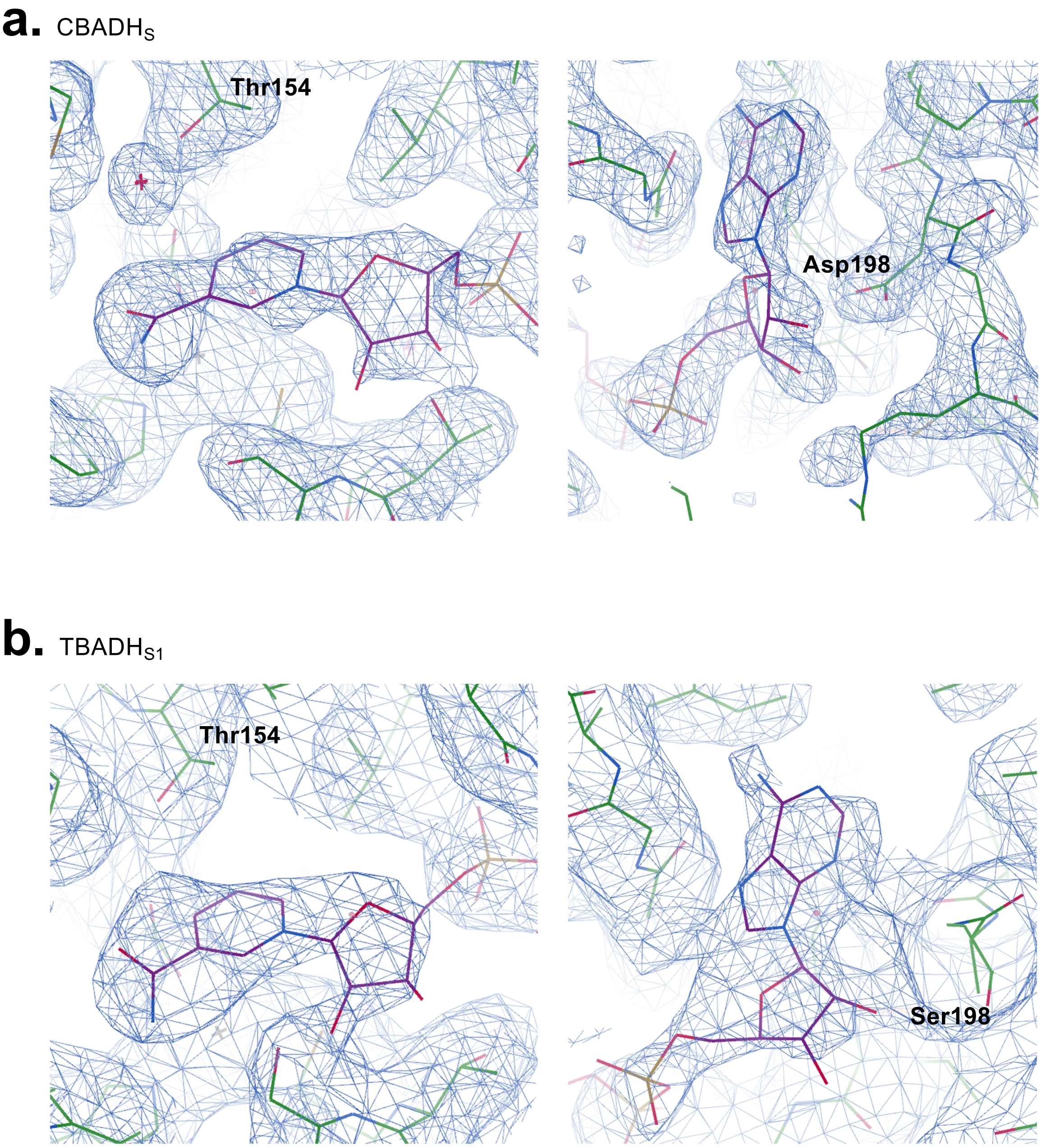


**Supplementary Figure 4. Density for the NAD$^+$ cofactor in the crystallographic maps of CBADH$_S$ and TBADH$_{S1}$.** Left panels display the density for the nicotinamide part of the cofactor, while right panels show the density for the adenine part. The density for the cofactor was stronger in CBADH$_S$ (**a**) than in TBADH$_{S1}$ (**b**), due to partial occupancy of the cofactor in the latter. 2F$_o$-F$_c$ maps are shown at sigma level of 1 for CBADH$_S$ and 0.8 for TBADH$_{S1}$. The NAD+ cofactors were refined with an approximate occupancy of 80%.

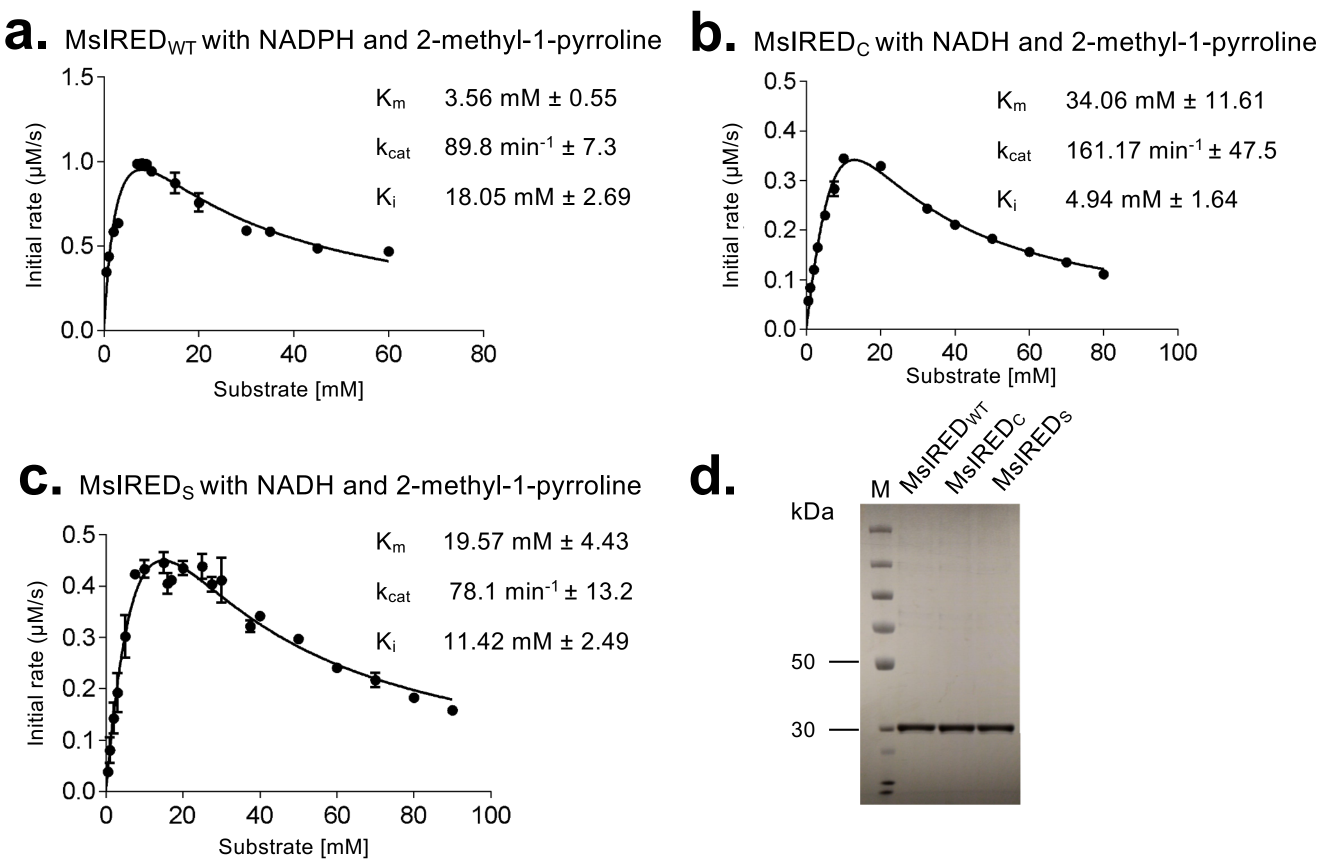


**Supplementary Figure 5. Enzymatic activity assays for MsIRED variants. a-c**, Initial reaction rates obtained for each enzyme by varying the 2-methyl-1-pyrroline concentration at a fixed concentration of NADH or NADPH. A Michaelis-Menten model equation modified to account for substrate inhibition was fitted in all cases. Data points represent mean values, with error bars showing standard deviation. n=3 biologically independent assays for all substrate concentrations with both enzymes. Source data are provided as a Source Data file. **d,** SDS-PAGE confirming the purity of wild-type MsIRED, MsIRED$_C$ and MsIRED$_S$ used for enzymatic assays. Active, pure proteins were successfully obtained with the described procedure from three independent preparations.

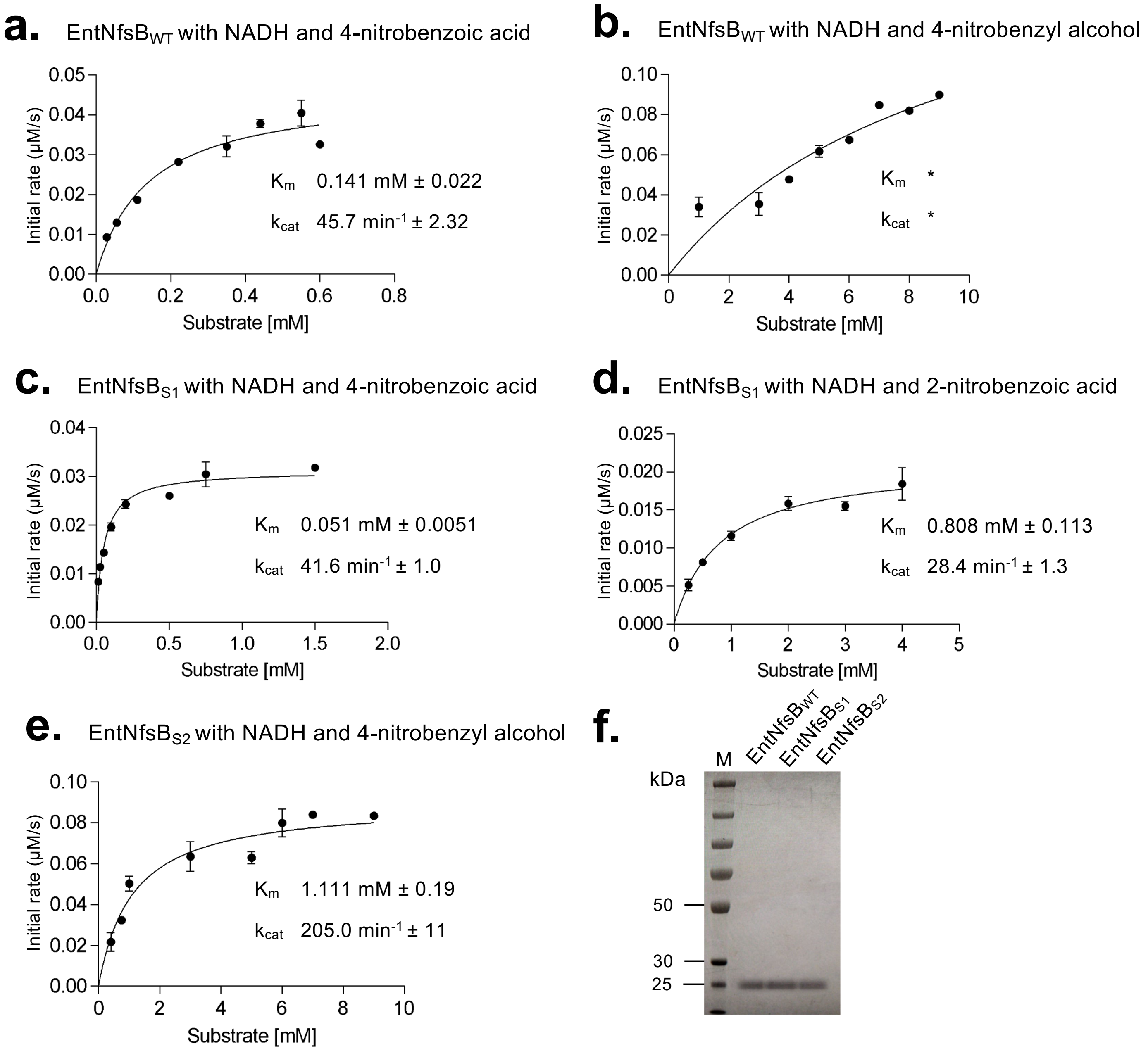


**Supplementary Figure 6. Enzymatic activity assays for EntNfsB variants. a-e**, Initial reaction rates obtained for each enzyme by varying nitroaromatic substrate (4-NBA, 2-NBA or 4-NBALC) concentration at a fixed concentration of NADH. A Michaelis-Menten model was fitted in all cases. Data points represent mean values, with error bars showing standard deviation. n=3 biologically independent assays for all substrate concentrations with both enzymes. Parameters that could not be determined due to a too low activity are indicated with an asterisk (*). Source data are provided as a Source Data file. **f,** SDS-PAGE confirming the purity of wild-type EntNfsB, EntNfsB$_{S1}$ and EntNfsB$_{S2}$ used for enzymatic assays. Active, pure proteins weresuccessfully obtained with the described procedure from three independent preparations.

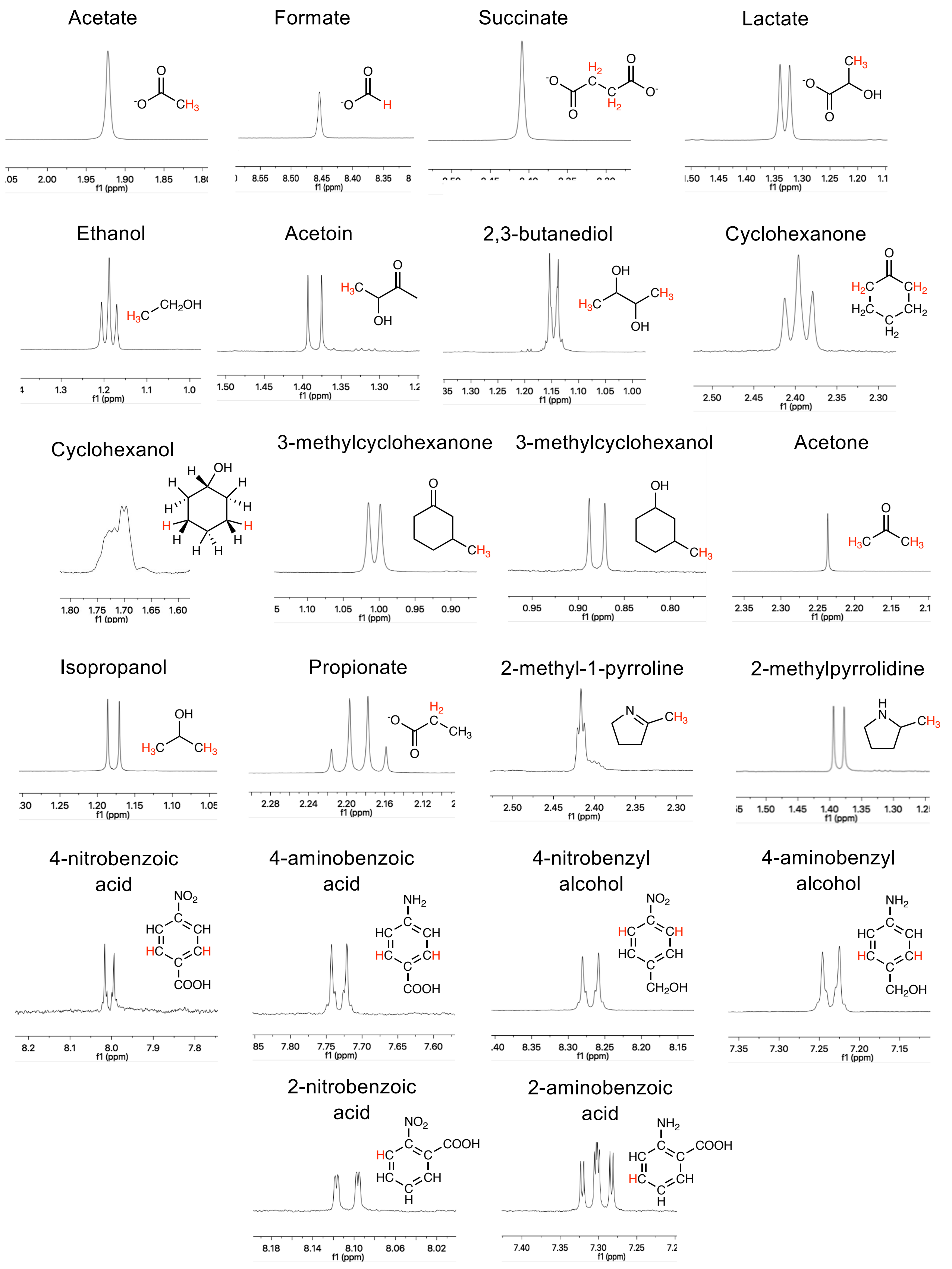


**Supplementary Figure 7. Characteristic $^{1}$H-NMR signals used to quantify each of the analyzed metabolites.** Protons contributing to the characteristic signal of each metabolite are highlighted in red.

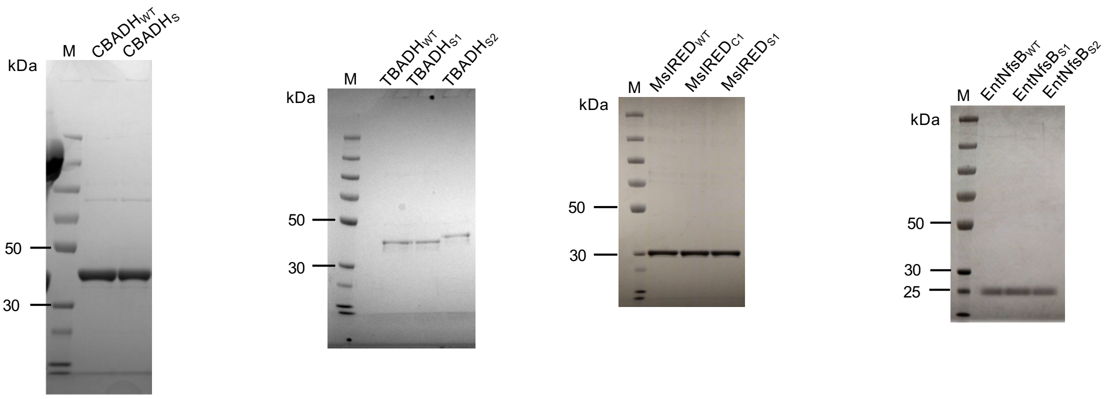


**Supplementary Figure 8. Uncropped gels.** The shown gels correspond, from left to right, to Supplementary Fig. 1g, Supplementary Fig. 1h, Supplementary Fig. 5d and Supplementary Fig. 6f.

**Supplementary Table 1. $^{1}$H-NMR analysis of fermentation broths.** Mean values and standard deviations are shown for each metabolite.

| Strain | Plasmid | Encoded enzyme | [Substrate] (mM) | [Product] (mM) | [Ethanol] (mM) | [Lactate] (mM) | [Succinate] (mM) | [Acetate] (mM) | [Formate] (mM) |
|---|---|---|---|---|---|---|---|---|---|
| **BW25113** | - | - | - | - | 15.4 ± 0.5 | 7.1 ± 0.4 | 2.3 ± 0.1 | 14.5 ± 0.5 | 26.2 ± 0.8 |
| **AL** | pLS1 | ADHE | - | - | 16.9 ± 0.4 | 0 | 2.1 ± 0.1 | 14.3 ± 0.3 | 25.6 ± 0.6 |
| **AL** | pUC19 | - | 3.2 ± 0.02 (Acetoin) | 5.9 ± 0.1 (2,3-butanediol) | 0 | 0 | 1.2 ± 0.03 | 6.7 ± 0.1 | 4.0 ± 0.1 |
| **AL** | pLS2 | BDHA | 0 (Acetoin) | 8.8 ± 0.2 (2,3-butanediol) | 0 | 0 | 0.6 ± 0.03 | 7.5 ± 0.3 | 4.5 ± 0.1 |
| **AL** | pLS3 | BUDC | 0.1 ± 0.002 (Acetoin) | 8.5 ± 0.1 (2,3-butanediol) | 0 | 0 | 0.7 ± 0.1 | 7.9 ± 0.2 | 5.7 ± 0.2 |
| **AL** | pLS12 | TADH | 0 (Cyclohexanone) | 9.1 ± 0.2 (Cyclohexanol) | 0 | 0 | 0.8 ± 0.05 | 9.9 ± 0.1 | 7.9 ± 0.2 |
| **AL** | pLS12 | TADH | 4.3 ± 0.1 (3-methylcyclohexanone) | 3.8 ± 0.1 (3-methylcyclohexanol) | 0 | 0 | 0.4 ± 0.01 | 3.7 ± 0.1 | 3.1 ± 0.04 |
| **AL** | pLS6 | $CBADH_{WT}$ | 0.2 ± 0.008 (Acetone) | 10.8 ± 0.2 (Isopropanol) | 0 | 0 | 1.2 ± 0.02 | 8.3 ± 0.2 | 6.1 ± 0.1 |
| **AL** | pLS10_3 | $CBADH_{S}$ | 0.6 ± 0.1 (Acetone) | 12.5 ± 0.8 (Isopropanol) | 0 | 0 | 1.3 ± 0.1 | 12.8 ± 0.9 | 9.9 ± 0.7 |
| **ALPS** | pLS69 | $TBADH_{WT}$ | 8.4 ± 0.2 (Acetone) | 0 (Isopropanol) | 0 | 0 | 0 | 0 | 0 |
| **ALPS** | pLS73_2 | $TBADH_{s1}$ | 0.1 ± 0.01 (Acetone) | 14.8 ± 0.3 (Isopropanol) | 0 | 0 | 1.6 ± 0.1 | 15.2 ± 0.4 | 11.2 ± 0.7 |
| **ALPS** | pLS73_1 | $TBADH_{s2}$ | 0.1 ± 0.04 (Acetone) | 15.4 ± 0.4 (Isopropanol) | 0 | 0 | 1.7 ± 0.1 | 17.2 ± 0.5 | 12.5 ± 0.3 |
| **AL** | pLS130 | $MsIRED_{WT}$ | 5.6 ± 0.1 (2-methyl-1-pyrroline) | 6.5 ± 0.1 (2-methylpyrrolidine) | 0 | 0 | 1.1 ± 0.1 | 4.3 ± 0.3 | 2.1 ± 0.1 |
| **AL** | pLS131 | $MsIRED_{C}$ | 3.3 ± 0.009 (2-methyl-1-pyrroline) | 9.3 ± 0.03 (2-methylpyrrolidine) | 0 | 0 | 1.7 ± 0.01 | 10.4 ± 0.2 | 8.5 ± 0.1 |
| **AL** | pLS133_1 | $MsIRED_{S}$ | 0.8 ± 0.1 (2-methyl-1-pyrroline) | 13.3 ± 0.2 (2-methylpyrrolidine) | 0 | 0 | 1.9 ± 0.1 | 15.6 ± 0.3 | 13.6 ± 0.7 |
| **AL** | pLS168 | $EntNfsB_{WT}$ | 0 (4-nitrobenzoic acid) | 0.4 ± 0.008 (4-aminobenzoic acid) | 0 | 0 | 0.3 ± 0.02 | 2.9 ± 0.1 | 2.3 ± 0.2 |

| | | | | | | | | | |
|---|---|---|---|---|---|---|---|---|---|
| **AL** | pLS168 | $EntNfsB_{WT}$ | 8.0 ± 0.02 (2-nitrobenzoic acid) | 0 (2-aminobenzoic acid) | 0 | 0 | 0 | 0 | 0 |
| **AL** | pLS168 | $EntNfsB_{WT}$ | 0.2 ± 0.02 (4-nitrobenzyl alcohol) | 0.5 ± 0.03 (4-aminobenzyl alcohol) | 0 | 0 | 0.5 ± 0.1 | 6.3 ± 0.1 | 5.8 ± 0.1 |
| **AL** | pLS169_1 | $EntNfsB_{s1}$ | 3.2 ± 0.4 (2-nitrobenzoic acid) | 3.5 ± 0.04 (2-aminobenzoic acid) | 0 | 0 | 1.2 ± 0.1 | 12 ± 0.4 | 8.9 ± 0.4 |
| **AL** | pLS169_3 | $EntNfsB_{s2}$ | 0 (4-nitrobenzyl alcohol) | 0.8 ± 0.3 (4-aminobenzyl alcohol) | 0 | 0 | 0.4 ± 0.1 | 6.1 ± 0.9 | 5.0 ± 1.2 |
| **BW25113** | pStA212 | - | 0 (Acetone) | 0 (Isopropanol) | 0 | 0 | 0 | 0 | 0 |
| **BW25113** | pLS60_1 | AtoB/AtoA/AtoD/ADC/CBA $DH_{WT}$ | 12.0 ± 3.6 (Acetone) | 62.3 ± 5.1 (Isopropanol) | 0 | 0.8 ± 0.3 | 2.6 ± 1.6 | 3.5 ± 1.7 | 3.6 ± 2.3 |

**Supplementary Table 2. Summary of kinetic parameters of wild-type and evolved enzymes.** Parameters that could not be determined due to a too low activity are indicated with an asterisk (*). Cases for which substrate inhibition was not observed are indicated with a hyphen (-).

| Enzyme | Substrate | Cosubstrate | $K_m$ | $k_{cat}$ ($min^{-1}$) | $k_{cat}/K_m$ | $K_i$ (mM) |
|---|---|---|---|---|---|---|
| **$CBADH_{WT}$** | Isopropanol | $NADP^+$ | 5.80 ± 0.25 mM | 1185.6 ± 14.0 | 204.4 ± 9.1 $min^{-1}mM^{-1}$ | - |
| **$CBADH_S$** | Isopropanol | $NAD^+$ | 17.49 ± 2.30 mM | 333.0 ± 12.8 | 19.0 ± 2.6 $min^{-1}mM^{-1}$ | - |
| **$TBADH_{WT}$** | Isopropanol | $NADP^+$ | 119.4 ± 6.9 mM | 498.0 ± 9.2 | 4.2 ± 0.25 $min^{-1}mM^{-1}$ | - |
| **$TBADH_{S1}$** | Isopropanol | $NAD^+$ | 3.74 ± 0.54 mM | 111.5 ± 5.7 | 29.8 ± 4.6 $min^{-1}mM^{-1}$ | - |
| **$TBADH_{S2}$** | Isopropanol | $NAD^+$ | 22.07 ± 2.70 mM | 238.5 ± 8.7 | 10.8 ± 1.4 $min^{-1}mM^{-1}$ | - |
| **$TBADH_{S2}$** | Isopropanol | $NADP^+$ | 55.15 ± 2.57 mM | 231.4 ± 3.7 | 4.2 ± 0.2 $min^{-1}mM^{-1}$ | - |
| **$MsIRED_{WT}$** | 2-methyl-1-pyrroline | NADPH | 3.56 ± 0.55 mM | 89.8 ± 7.3 | - | 18.05 ± 2.69 |
| **$MsIRED_C$** | 2-methyl-1-pyrroline | NADH | 34.06 ± 11.61 mM | 161.2 ± 47.5 | - | 4.94 ± 1.64 |
| **$MsIRED_S$** | 2-methyl-1-pyrroline | NADH | 19.57 ± 4.43 mM | 78.1 ± 13.2 | - | 11.42 ± 2.49 |
| **$EntNfsB_{WT}$** | 4-nitrobenzoic acid | NADH | 0.141 ± 0.022 mM | 45.7 ± 2.3 | 324.1 ± 53.2 $min^{-1}mM^{-1}$ | - |
| **$EntNfsB_{WT}$** | 4-nitrobenzyl alcohol | NADH | * | * | * | - |
| **$EntNfsB_{S1}$** | 4-nitrobenzoic acid | NADH | 0.051 ± 0.0051 mM | 41.6 ± 1.0 | 815.7 ± 83.9 $min^{-1}mM^{-1}$ | - |
| **$EntNfsB_{S1}$** | 2-nitrobenzoic acid | NADH | 0.81 ± 0.113 mM | 28.4 ± 1.3 | 35.1 ± 5.1 $min^{-1}mM^{-1}$ | - |
| **$EntNfsB_{S2}$** | 4-nitrobenzyl alcohol | NADH | 1.11 ± 0.19 mM | 205.0 ± 11.0 | 184.5 ± 33.1 $min^{-1}mM^{-1}$ | - |
| **$CBADH_{WT}$** | $NADP^+$ | Isopropanol | 55.91 ± 6.58 μM | 1072.5 ± 55.17 | 19.18 ± 2.46 $min^{-1}\mu M^{-1}$ | - |
| **$CBADH_S$** | $NAD^+$ | Isopropanol | 934.4 ± 136.2 μM | 290.58 ± 23.17 | 0.31 ± 0.05 $min^{-1}\mu M^{-1}$ | - |
| **$TBADH_{WT}$** | $NADP^+$ | Isopropanol | 56.67 ± 11.63 μM | 482.19 ± 25.9 | 8.51 ± 1.81 $min^{-1}\mu M^{-1}$ | - |
| **$TBADH_{S1}$** | $NAD^+$ | Isopropanol | 1.03 ± 0.16 mM | 109.0 ± 4.0 | 105.6 ± 17.1 $min^{-1}mM^{-1}$ | - |
| **$TBADH_{S2}$** | $NAD^+$ | Isopropanol | 104.4 ± 9.68 μM | 220.12 ± 6.17 | 2.11 ± 0.20 $min^{-1}\mu M^{-1}$ | - |
| **$TBADH_{S2}$** | $NADP^+$ | Isopropanol | 240.7 ± 29.4 μM | 234.68 ± 10.57 | 0.97 ± 0.13 $min^{-1}\mu M^{-1}$ | - |

| | | | | | | |
|---|---|---|---|---|---|---|
| **MsIRED$_{WT}$** | NADPH | 2-methyl-1-pyrroline | 24.67 ± 3.68 μM | 56.65 ± 2.9 | 2.30 ± 0.36 $min^{-1}μM^{-1}$ | - |
| **MsIRED$_{C}$** | NADH | 2-methyl-1-pyrroline | 23.66 ± 2.5 μM | 15.05 ± 0.79 | 0.64 ± 0.075 $min^{-1}μM^{-1}$ | - |
| **MsIRED$_{S}$** | NADH | 2-methyl-1-pyrroline | 19.9 ± 1.71 μM | 21.10 ± 0.84 | 1.06 ± 0.1 $min^{-1}μM^{-1}$ | - |
| **EntNfsB$_{WT}$** | NADH | 4-nitrobenzoic acid | 29.78 ± 1.3 μM | 46.48 ± 1.03 | 1.56 ± 0.076 $min^{-1}μM^{-1}$ | - |
| **EntNfsB$_{WT}$** | NADH | 4-nitrobenzyl alcohol | 30.65 ± 3.96 μM | 297.7 ± 18.23 | 9.71 ± 1.39 $min^{-1}μM^{-1}$ | - |
| **EntNfsB$_{S1}$** | NADH | 4-nitrobenzoic acid | 28.90 ± 1.87 μM | 41.88 ± 1.14 | 1.45 ± 0.10 $min^{-1}μM^{-1}$ | - |
| **EntNfsB$_{S1}$** | NADH | 2-nitrobenzoic acid | 26.37 ± 3.49 μM | 28.57 ± 1.20 | 1.08 ± 0.15 $min^{-1}μM^{-1}$ | - |
| **EntNfsB$_{S2}$** | NADH | 4-nitrobenzyl alcohol | 28.86 ± 2.73 μM | 219.16 ± 9.0 | 7.59 ± 0.78 $min^{-1}μM^{-1}$ | - |

**Supplementary Table 3. Plasmids used in this work.**

| Plasmid | Description | Antibiotic resistance | Source |
|---|---|---|---|
| **pUC19** | High copy expression vector (pMB1 origin of replication) with lacZα – **Negative control** for anaerobic growth complementation experiments | AmpR | Heap Laboratory (Norrander, Kempe, and Messing, 1983) |
| **pJET1.2** | Cloning vector | AmpR | Invitrogen |
| **pET28a** | Bacterial expression vector with 6xHis-tag | KanR | Invitrogen |
| **pMAK705** | *E. coli* vector for directed mutagenesis by allele exchange | CmR | Heap Laboratory (Hamilton, *et al.*, 1989) |
| **pStA0** | Start-Stop Assembly Level 0 storage vector | AmpR | Heap Laboratory (Taylor *et al.*, 2019) |
| **pStA1AB** | Start-Stop Assembly Level 1 vector (A and B fusion sites) | TetR | Heap Laboratory (Taylor *et al.*, 2019) |
| **pStA1BC** | Start-Stop Assembly Level 1 vector (B and C fusion sites) | TetR | Heap Laboratory (Taylor *et al.*, 2019) |
| **pStA1CD** | Start-Stop Assembly Level 1 vector (C and D fusion sites) | TetR | Heap Laboratory (Taylor *et al.*, 2019) |
| **pStA1DE** | Start-Stop Assembly Level 1 vector (D and E fusion sites) | TetR | Heap Laboratory (Taylor *et al.*, 2019) |
| **pStA1EZ** | Start-Stop Assembly Level 1 vector (E and Z fusion sites) | TetR | Heap Laboratory (Taylor *et al.*, 2019) |
| **pGT323** | pStA0 containing BBa_J23100 promoter | AmpR | Heap Laboratory (Taylor *et al.*, 2019) |
| **pGT324** | pStA0 containing BBa_J23102 promoter | AmpR | Heap Laboratory (Taylor *et al.*, 2019) |
| **pGT326** | pStA0 containing BBa_J23107 promoter | AmpR | Heap Laboratory (Taylor *et al.*, 2019) |
| **pGT327** | pStA0 containing BBa_J23116 promoter | AmpR | Heap Laboratory (Taylor *et al.*, 2019) |
| **pGT328** | pStA0 containing BBa_J23113 promoter | AmpR | Heap Laboratory (Taylor *et al.*, 2019) |
| **pGT336** | pStA0 containing BBa_J23118 promoter | AmpR | Heap Laboratory (Taylor *et al.*, 2019) |
| **pGT330** | pStA0 containing RBSc13 | AmpR | Heap Laboratory (Taylor *et al.*, 2019) |
| **pGT331** | pStA0 containing RBSc33 | AmpR | Heap Laboratory (Taylor *et al.*, 2019) |
| **pGT332** | pStA0 containing RBSc44 | AmpR | Heap Laboratory (Taylor *et al.*, 2019) |
| **pGT333** | pStA0 containing RBSc58 | AmpR | Heap Laboratory (Taylor *et al.*, 2019) |
| **pGT334** | pStA0 containing RBSc36 | AmpR | Heap Laboratory (Taylor *et al.*, 2019) |

| | | | |
|---|---|---|---|
| **pGT335** | pStA0 containing RBSc42 | AmpR | Heap Laboratory (Taylor *et al.*, 2019) |
| **pGT337** | pStA0 containing Terminator 1 (L3S2P55) | AmpR | Heap Laboratory (Taylor *et al.*, 2019) |
| **pGT338** | pStA0 containing Terminator 2 (L3S2P21) | AmpR | Heap Laboratory (Taylor *et al.*, 2019) |
| **pGT339** | pStA0 containing Terminator 3 (ECK120033737) | AmpR | Heap Laboratory (Taylor *et al.*, 2019) |
| **pGT340** | pStA0 containing Terminator 4 (ECK1200196000) | AmpR | Heap Laboratory (Taylor *et al.*, 2019) |
| **pStA212** | Start-Stop Assembly Level 2 vector (fusion sites 1 and 2) – **Negative control** for isopropanol pathway | KanR | Heap Laboratory (Taylor *et al.*, 2019) |
| **pLS1** | pUC19 containing sequence encoding ADHE of *E. coli* BW25113 (AIN31697.1) – **Positive control** for anaerobic growth experiments | AmpR | This work |
| **pLS2** | pUC19 containing sequence encoding BDHA of *Bacillus subtilis* 168 (CAB12443.1) | AmpR | This work |
| **pLS3_1** | pJET1.2 storage plasmid containing sequence encoding BUDC of *Klebsiella pneumoniae* (AAC78679.1) | AmpR | This work |
| **pLS3** | pUC19 containing sequence encoding BUDC of *Klebsiella pneumoniae* (AAC78679.1) | AmpR | This work |
| **pLS6** | pUC19 containing sequence encoding CBADH of *Clostridium beijerinckii* (AAA23199.2) | AmpR | This work |
| **pLS10** | Library equivalent to pLS6 with saturation mutagenesis of amino acid positions 198, 199, 200 and 218 of CBADH ($CBADH_{Lib}$) | AmpR | This work |
| **pLS10_3** | Plasmid from library pLS10 encoding variant $CBADH_S$ (G198D, S199Y, R200R, Y218P) | AmpR | This work |
| **pLS12_1** | pJET1.2 storage plasmid containing sequence encoding TADH of *Thermus* sp. ATN-1 (ACD50896.1) | AmpR | This work |

| | | | |
|---|---|---|---|
| **pLS12** | pUC19 containing sequence encoding TADH of *Thermus* sp. ATN-1 (ACD50896.1) | AmpR | This work |
| **pLS39** | pMAK705 with 500 bp upstream and 500 bp downstream of *sthA* from *E. coli* | CmR | This work |
| **pLS40** | pMAK705 with 500 bp upstream and 500 bp downstream of *pntA* from *E. coli* | CmR | This work |
| **pLS46** | pStA0 containing *atoB* gene of *E. coli* (AAC75284.1) | AmpR | This work |
| **pLS47** | pStA0 containing *atoD* gene of *E. coli* (AAC75281.1) | AmpR | This work |
| **pLS48** | pStA0 containing *atoA* gene of *E. coli* (AAC75282.1) | AmpR | This work |
| **pLS49** | pStA0 containing *adc* gene of *Clostridium acetobutylicum* (AAA63761.1) | AmpR | This work |
| **pLS50** | pStA0 containing *cbadh* gene of *Clostridium beijerinckii* (AAA23199.2) | AmpR | This work |
| **pLS53** | pStA1AB containing full transcription unit coding for acetyl-CoA acetyltransferase from *E. coli* (*atoB*) (library of six promoters and six RBS, Terminator 1) | TetR | This work |
| **pLS54** | pStA1BC containing full transcription unit coding for acetate CoA-transferase subunit alpha from *E. coli* (*atoD*) (library of six promoters and six RBS, Terminator 2) | TetR | This work |
| **pLS55** | pStA1CD containing full transcription unit coding for acetate CoA-transferase subunit beta from *E. coli* (*atoA*) (library of six promoters and six RBS, Terminator 3) | TetR | This work |
| **pLS56** | pStA1DE containing full transcription unit coding for acetoacetate decarboxylase from *Clostridium acetobutylicum* (*adc*) (library of six promoters and six RBS, Terminator 4) | TetR | This work |

| | | | |
|---|---|---|---|
| **pLS57** | pStA1EZ containing full transcription unit coding for NADP-dependent isopropanol dehydrogenase from *Clostridium beijerinckii* (*cbadh*) (library of six promoters and six RBS, Terminator 1) | TetR | This work |
| **pLS60** | pStA212 containing combinatorial isopropanol pathway library $MP_{Lib}$ | KanR | This work |
| **pLS60_1** | Plasmid from library $MP_{Lib}$ (pLS60) encoding isopropanol pathway variant $MP_{S1}$ | KanR | This work |
| **pLS60_2** | Pathway from library $MP_{Lib}$ (pLS60) encoding isopropanol pathway variant $MP_{S2}$ | KanR | This work |
| **pLS63** | pMAK705 with 500 bp upstream and 500 bp downstream of *ldhA* from *E. coli* | CmR | This work |
| **pLS67** | pJET1.2 storage plasmid containing sequence encoding TBADH of *Thermoanaerobacter brockii* (CAA46053.1) | AmpR | This work |
| **pLS69** | pUC19 containing sequence encoding TBADH of *Thermoanaerobacter brockii* (CAA46053.1) | AmpR | This work |
| **pLS73** | Library equivalent to pLS69 with saturation mutagenesis of amino acid positions 198, 199, 200 and 218 of TBADH ($TBADH_{Lib}$) | AmpR | This work |
| **pLS73_1** | Plasmid from library pLS73 encoding variant $TBADH_{S2}$ (G198H, S199R, R200A, Y218M and a 153 bp insertion between residues 241 and 242) | AmpR | This work |
| **pLS73_2** | Plasmid from library pLS73 encoding variant $TBADH_{S1}$ (G198S, S199K, R200P, Y218V) | AmpR | This work |
| **pLS90** | pET28a containing sequence encoding $TBADH_{S2}$ with N-terminal 6xHis-tag | KanR | This work |

| | | | |
|---|---|---|---|
| **pLS91** | pET28a containing sequence encoding $TBADH_{S1}$ with N-terminal 6xHis-tag | KanR | This work |
| **pLS97** | pET28a containing sequence encoding wild-type TBADH with N-terminal 6xHis-tag | KanR | This work |
| **pLS98** | pET28a containing sequence encoding $CBADH_S$ with N-terminal 6xHis-tag | KanR | This work |
| **pLS99** | pET28a containing gene encoding wild-type CBADH with N-terminal 6xHis-tag | KanR | This work |
| **pLS129** | pJET1.2 storage plasmid containing sequence encoding MsIRED of *Myxococcus stipitatus* (AGC43099.1) | AmpR | This work |
| **pLS130** | pUC19 containing sequence encoding MsIRED of *Myxococcus stipitatus* (AGC43099.1) | AmpR | This work |
| **pLS131** | pUC19 containing sequence encoding $MsIRED_C$ variant of *Myxococcus stipitatus* MsIRED (N32E, R33Y, T34E, K37R, L67I, T71V) | AmpR | This work |
| **pLS133** | Library equivalent to pLS131 with saturation mutagenesis of amino acid positions 32, 33, 34 and 37 of MsIRED ($MsIRED_{Lib}$) | AmpR | This work |
| **pLS133_1** | Plasmid from library pLS133 encoding variant $MsIRED_S$ (N32E, R33V, T34R, K37R) | AmpR | This work |
| **pLS161** | pUC19 containing sequence encoding wild-type MsIRED with C-terminal 6xHis-tag | AmpR | This work |
| **pLS162** | pUC19 containing sequence encoding $MsIRED_C$ with C-terminal 6xHis-tag | AmpR | This work |
| **pLS164** | pUC19 containing sequence encoding $MsIRED_S$ with C-terminal 6xHis-tag | AmpR | This work |
| **pLS168_1** | pJET1.2 storage plasmid containing sequence encoding EntNfsB of *Enterobacter cloacae* (AAA62801.1) | AmpR | This work |
| **pLS168** | pUC19 containing sequence encoding EntNfsB of | AmpR | This work |

| | | | |
|---|---|---|---|
| | *Enterobacter cloacae* (AAA62801.1) | | |
| **pLS169** | Library equivalent to pLS168 with saturation mutagenesis of amino acid positions 40, 41, 68 and 124 of EntNfsB ($EntNfsB_{Lib}$) | AmpR | This work |
| **pLS169_1** | Plasmid from library pLS133 encoding variant $EntNfsB_{S1}$ (S40A, T41I, Y68Y, F124A) | AmpR | This work |
| **pLS169_3** | Plasmid from library pLS133 encoding variant $EntNfsB_{S2}$ (S40S, T41L, Y68L, F124L) | AmpR | This work |
| **pLS180** | pUC19 containing sequence encoding EntNfsB with C-terminal 6xHis-tag | AmpR | This work |
| **pLS181** | pUC19 containing sequence encoding $EntNfsB_{S1}$ with C-terminal 6xHis-tag | AmpR | This work |
| **pLS182** | pUC19 containing sequence encoding $EntNfsB_{S2}$ with C-terminal 6xHis-tag | AmpR | This work |

**Supplementary Table 4. Oligonucleotides used in this work.** Restriction sites are highlighted in green.

| Purpose | Template | Oligo ID | Description | Sequence (5' – 3') |
|---|---|---|---|---|
| Amplification of pUC19 for Golden Gate Assembly | pUC19 | oligoLS315 | Fw - *BbsI* | TCTCTGAAGACCCTAAGGATCCCCGGGTACC |
| Amplification of pUC19 for Golden Gate Assembly | pUC19 | oligoLS314 | Rv - *BbsI* | TCTCTGAAGACTCCATGTGTTCGTACCTCCTGCATG |
| Construction of pLS1 | *adhE* of *E. coli* BW25113 | oligoLS19 | Fw - *SphI* | CCGTTCGCATGCAGGAGGTACGAACACATGGCTGTTACTAATGT |
| Construction of pLS1 | *adhE* of *E. coli* BW25113 | oligoLS20 | Rv - *BamHI* | GCTGAAGGATCCTTAAGCGGATTTTTTCG |
| Verification of *adhE* KO | *adhE* of *E. coli* BW25113 | oligoLS1 | Fw - internal | CCTGTGGTGTTCTGTCTG |
| Verification of *adhE* KO | *adhE* of *E. coli* BW25113 | oligoLS2 | Rv - internal | TAGATTTCGGAATACCCA |
| Verification of *adhE* KO | *adhE* of *E. coli* BW25113 | oligoLS3 | Fw - external | GGCGAAAAGCGATGCTG |
| Verification of *adhE* KO | *adhE* of *E. coli* BW25113 | oligoLS4 | Rv - external | CGGTGGGAAGGTGTTCTGC |
| Verification of *ldhA* KO | *ldhA* of *E. coli* BW25113 | oligoLS5 | Fw - internal | GCCGCCCGGTGCTGGAAG |
| Verification of *ldhA* KO | *ldhA* of *E. coli* BW25113 | oligoLS6 | Rv - internal | GGCGACGGAATACGTCAT |
| Verification of *ldhA* KO | *ldhA* of *E. coli* BW25113 | oligoLS7 | Fw - external | GAAGGTTGCGCCTACACT |
| Verification of *ldhA* KO | *ldhA* of *E. coli* BW25113 | oligoLS8 | Rv - external | CACCAAAGCTGATTTCTG |
| Construction of pLS2 | Sequence encoding BDHA of *Bacillus subtilis* 168 | oligoLS23 | Fw - *SphI* | CCGTTCGCATGCAGGAGGTACGAACACATGAAGGCAGCAAGATG |
| Construction of pLS2 | Sequence encoding BDHA of *Bacillus subtilis* 168 | oligoLS24 | Rv - *BamHI* | GCTGAAGGATCCTTAGTTAGGTCTAACAAGGATTTTGACT |
| Construction of pLS6 | Sequence encoding CBADH of *Clostridium beijerinckii* | oligoLS87 | Fw - *SphI* | GTTCGCATGCATTCGGATCTATACAGATAAGGAGAAAGAGATGAAAGGCTTTGCCATGCTGGG |
| Construction of pLS6 | Sequence encoding CBADH of *Clostridium beijerinckii* | oligoLS88 | Rv - *BamHI* | CTTCCATGGATCCTCACTATTAGAGGATAACTACGGCC |
| Construction of pLS10 library ($CBADH_{Lib}$) | Sequence encoding CBADH of *Clostridium beijerinckii* | oligoLS112 | Rv | CTTGGCGGCCTCAACGCAAATAGGNNNNNNNNNGACACCAATAATCCGACCTGC |

| | | | | |
|---|---|---|---|---|
| Construction of pLS10 library (CBADH$_{Lib}$) | Sequence encoding CBADH of *Clostridium beijerinckii* | oligoLS113 | Fw | TTCTACGGCGCGACCGACATTCTGAATNNNAAAAATGGCCATATTGTGGAC |
| Construction of pLS39 | 500 bp upstream and downstream of *sthA* | oligoLS208 | Fw - *BamHI* | TTCAGCGGATCCAATGTATCTGCATGAAGCACAGACCCACCAGTTACTGG |
| Construction of pLS39 | 500 bp upstream and downstream of *sthA* | oligoLS212 | Rv | AACAGGTAAGCCCTACCATGTAAAACTTTATCGAAATGGCCATCCATTCTTGCGCGG |
| Construction of pLS39 | 500 bp upstream and downstream of *sthA* | oligoLS213 | Fw | GCCATTTCGATAAAGTTTTACATGGTAGGGCTTACCTGTTCTTATACATAAAAGCAACAGAATGG |
| Construction of pLS39 | 500 bp upstream and downstream of *sthA* | oligoLS209 | Rv - *HindIII* | TTCAGCAAGCTTCATTAAACCGCTCTCATCAACCATGGTCAGACCCAGTTCG |
| Verification of *sthA* KO | *sthA* of *E. coli* BW25113 | oligoLS194 | Fw - internal | GATGGAACAAAATTTTCAGCGTGCC |
| Verification of *sthA* KO | *sthA* of *E. coli* BW25113 | oligoLS193 | Rv - internal | ATAGTAATAGGTTCCGGCCC |
| Verification of *sthA* KO | *sthA* of *E. coli* BW25113 | oligoLS195 | Fw - external | CAGGCAATGGGTTTCTGTTTTG |
| Verification of *sthA* KO | *sthA* of *E. coli* BW25113 | oligoLS196 | Rv - external | CGAACTGGGTCTGACCATGGTTGATGAGAGCGGTTTAATG |
| Construction of pLS40 | 500 bp upstream and downstream of *pntA* | oligoLS216 | Fw - *BamHI* | TTCAGCGGATCCGAAACGACCAGAGCCGCCAGGTTCA |
| Construction of pLS40 | 500 bp upstream and downstream of *pntA* | oligoLS217 | Rv | CCGATGGAAGGGAATATCATGTAAGGGGTAACATATGTCTGGAGGATTAGTTACAGCTGCATACATTGTTGCCGC |
| Construction of pLS40 | 500 bp upstream and downstream of *pntA* | oligoLS219 | Fw | CCAGACATATGTTACCCCTTACATGATATTCCCTTCCATCGGTTTTATTGATG |
| Construction of pLS40 | 500 bp upstream and downstream of *pntA* | oligoLS218 | Rv - *HindIII* | TTCAGCAAGCTTCAGGAGGGTGTTCTTAAGCTTCATAAAAATAATCCTTCGCCTTGCGCAAA |
| Verification of *pntA* KO | *pntA* of *E. coli* BW25113 | oligoLS223 | Fw - internal | GTGCTCCGACAACAATAATCC |
| Verification of *pntA* KO | *pntA* of *E. coli* BW25113 | oligoLS224 | Rv - internal | TGATGGTGATTGGTGCGGGTG |
| Verification of *pntA* KO | *pntA* of *E. coli* BW25113 | oligoLS216 | Fw - external | GAAACGACCAGAGCCGCCAGGTTCA |
| Verification of *pntA* KO | *pntA* of *E. coli* BW25113 | oligoLS221 | Rv - external | TTTGCGCAAGGCGAAGGATTATTTTTATGAAGC |
| Construction of pLS46 | *atoB* of *E. coli* BW25113 | oligoLS230 | Fw - *BsaI* | AAGGGGTTGGTCTCATGTGCTCTTCGATGAAAAATTGTGTCATCGTCAGTGCGGTACG |
| Construction of pLS46 | *atoB* of *E. coli* BW25113 | oligoLS231 | Rv - *BsaI* | AAGGGGTTGGTCTCTGGTCTTACGCTCTTCATTAATTCAACCGTTCAATCACCATCGCAATTCCC |
| Construction of pLS47 | *atoD* of *E. coli* BW25113 | oligoLS234 | Fw - *BsaI* | AAGGGGTTGGTCTCATGTGGCTCTTCGATGAAAACAAAATTGATGACATTACAAGACG |
| Construction of pLS47 | *atoD* of *E. coli* BW25113 | oligoLS243 | Rv - *BsaI* | AAGGGGTTGGTCTCTGGTCTTACGCTCTTCATTATTTGCTCTCCTGTGAAACGATGATGTG |
| Construction of pLS48 | *atoA* of *E. coli* BW25113 | oligoLS235 | Fw - *BsaI* | AAGGGGTTGGTCTCTGGTCTTACGCTCTTCATTATAAATCACCCCGTTGCGTATTC |
| Construction of pLS48 | *atoA* of *E. coli* BW25113 | oligoLS242 | Rv - *BsaI* | AAGGGGTTGGTCTCATGTGGCTCTTCGATGGATGCGAAACAACGTATTGCGC |

| | | | | |
|---|---|---|---|---|
| Construction of pLS49 | *adc* of *Clostridium acetobutylicum* ATCC 824 | oligoLS228 | Fw - *BsaI* | AAGGGGTTGGTCTCATGTGGCTCTTCGATGTTAAAGGATGAAGTAATTAAACAAATTAGCACG |
| Construction of pLS49 | *adc* of *Clostridium acetobutylicum* ATCC 824 | oligoLS229 | Rv - *BsaI* | AAGGGGTTGGTCTCTGGTCTTACGCTCTTCATTACTTAAGATAATCATATATAACTTCAGCTCTAGGC |
| Construction of pLS50 | Sequence encoding CBADH of *Clostridium beijerinckii* | oligoLS232 | Fw - *BsaI* | AAGGGGTTGGTCTCATGTGGCTCTTCGATGAAAGGCTTTGCCATGCTGGGTATTAAC |
| Construction of pLS50 | Sequence encoding CBADH of *Clostridium beijerinckii* | oligoLS233 | Rv - *BsaI* | AAGGGGTTGGTCTCTGGTCTTACGCTCTTCATTAGAGGATAACTACGGCCTTAATGAGATCTTTAGG |
| Construction of pLS63 | 500 bp upstream and downstream of *ldhA* | oligoLS244 | Fw - *BamHI* | TTCAGCGGATCCTGTCTGTTTTGCGGTCGCCAG |
| Construction of pLS63 | 500 bp upstream and downstream of *ldhA* | oligoLS247 | Rv | CACTGGAGAAAGTCTTATGTAATCTTGCCGCTCCCCTGCATTCCAG |
| Construction of pLS63 | 500 bp upstream and downstream of *ldhA* | oligoLS246 | Fw | CAGGGGAGCGGCAAGATTACATAAGACTTTCTCCAGTGATGTTGAATC |
| Construction of pLS63 | 500 bp upstream and downstream of *ldhA* | oligoLS245 | Rv - *HindIII* | TTCAGCAAGCTTCAAGCAGAATCAAGTTCTACCGTGC |
| Construction of pLS73 library ($TBADH_{Lib}$) | Sequence encoding TBADH of *Thermoanaerobacter brockii* | oligoLS258 | Rv | AGCGTCGACACAGACTGGNNNNNNNNNAACAGCAATGATACGTCCTGC |
| Construction of pLS73 library ($TBADH_{Lib}$) | Sequence encoding TBADH of *Thermoanaerobacter brockii* | oligoLS259 | Fw | GCCAAGTATTACGGAGCAACCGACATCGTGAACNNNAAGGATGGGCC |
| Construction of pLS90 | Sequence encoding $TBADH_{S2}$ | oligoLS288 | Fw - *NdeI* | GCAGCCATATGATGAAGGGGTTCGCAATGCTGTCTATTGG |
| Construction of pLS90 | Sequence encoding $TBADH_{S2}$ | oligoLS289 | Rv - *BlpI* | TTATTGCTCAGCTTAAGCCAGAATAACCACTGGTTTGATAAGGTCCTTTGG |
| Construction of pLS91 | Sequence encoding $TBADH_{S2}$ | oligoLS288 | Fw - *NdeI* | GCAGCCATATGATGAAGGGGTTCGCAATGCTGTCTATTGG |
| Construction of pLS91 | Sequence encoding $TBADH_{S2}$ | oligoLS289 | Rv - *BlpI* | TTATTGCTCAGCTTAAGCCAGAATAACCACTGGTTTGATAAGGTCCTTTGG |
| Construction of pLS97 | Sequence encoding TBADH of *Thermoanaerobacter brockii* | oligoLS288 | Fw - *NdeI* | GCAGCCATATGATGAAGGGGTTCGCAATGCTGTCTATTGG |
| Construction of pLS97 | Sequence encoding TBADH of *Thermoanaerobacter brockii* | oligoLS289 | Rv - *BlpI* | TTATTGCTCAGCTTAAGCCAGAATAACCACTGGTTTGATAAGGTCCTTTGG |
| Construction of pLS98 | Sequence encoding $CBADH_{S}$ | oligoLS294 | Fw - *NdeI* | GCAGCCATATGATGAAAGGCTTTGCCATGCTGGGTATTAACAAATTAGG |
| Construction of pLS98 | Sequence encoding $CBADH_{S}$ | oligoLS295 | Rv - *BlpI* | TTATTGCTCAGCTTAGAGGATAACTACGGCCTTAATGAGATCTTTAGGTTTATCTTTCATGAG |

| | | | | |
|---|---|---|---|---|
| Construction of pLS99 | Sequence encoding CBADH of *Clostridium beijerinckii* | oligoLS294 | Fw - *NdeI* | GCAGCCATATGATGAAAGGCTTTGCCATGCTGGGTATTAACAAATTAGG |
| Construction of pLS99 | Sequence encoding CBADH of *Clostridium beijerinckii* | oligoLS295 | Rv - *BlpI* | TTATTGCTCAGCTTAGAGGATAACTACGGCCTTAATGAGATCTTTAGGTTTATCTTTCATGAG |
| Construction of pLS131 | Sequence encoding $MsIRED_C$ | oligoLS344 | Rv | ACGATAATATCGCTGCGTTTAAC |
| Construction of pLS131 | Sequence encoding $MsIRED_C$ | oligoLS345 | Fw | CTGGCAAAACTGGGCGCACATC |
| Construction of pLS131 | Sequence encoding $MsIRED_C$ | oligoLS342 | Rv | /5'Phos/CGGTTCGCTACGGGCTTTTTCATATTCCCACACCGTGGTCG |
| Construction of pLS131 | Sequence encoding $MsIRED_C$ | oligoLS343 | Fw | /5'Phos/GGTTAATGTGATTGATTATGACGTTTCTGATCAGCTGCTG |
| Construction of pLS133 library ($MsIRED_{Lib}$) | Sequence encoding MsIRED of *Myxococcus stipitatus* | oligoLS337 | Fw | GCTGAGAAGACCGACCACGGTGTGGNNNNNNNNNAAAGCCNNNAGCGAACCGCTGGCAAAACTG |
| Construction of pLS133 library ($MsIRED_{Lib}$) | Sequence encoding MsIRED of *Myxococcus stipitatus* | oligoLS338 | Rv | GCTGAGAAGACCGTGGTCGTGTAGCCAGATTGCAGGAATGCTTTAATCAGTGCGGAGCCCATACGGCC |
| Construction of pLS161 | Sequence encoding wild-type MsIRED with C-terminal 6xHis-tag | oligoLS358 | Rv - *BbsI* 6xHis-tag | TCTCTGAAGACTCCTTAGTGGTGGTGGTGGTGGTGTTTCAGGAAGCGGGTCAGAATTGCAAAG |
| Construction of pLS161 | Sequence encoding wild-type MsIRED with C-terminal 6xHis-tag | oligoLS359 | Fw - *BbsI* | TCTCTGAAGACAACATGAAACCGACCCTGACCGTTATTGGC |
| Construction of pLS162 | Sequence encoding $MsIRED_C$ with C-terminal 6xHis-tag | oligoLS358 | Rv - *BbsI* 6xHis-tag | TCTCTGAAGACTCCTTAGTGGTGGTGGTGGTGGTGTTTCAGGAAGCGGGTCAGAATTGCAAAG |
| Construction of pLS162 | Sequence encoding $MsIRED_C$ with C-terminal 6xHis-tag | oligoLS359 | Fw- *BbsI* | TCTCTGAAGACAACATGAAACCGACCCTGACCGTTATTGGC |
| Construction of pLS164 | Sequence encoding $MsIRED_S$ with C-terminal 6xHis-tag | oligoLS358 | Rv - *BbsI* 6xHis-tag | TCTCTGAAGACTCCTTAGTGGTGGTGGTGGTGGTGTTTCAGGAAGCGGGTCAGAATTGCAAAG |
| Construction of pLS164 | Sequence encoding $MsIRED_S$ with C-terminal 6xHis-tag | oligoLS359 | Fw - *BbsI* | TCTCTGAAGACAACATGAAACCGACCCTGACCGTTATTGGC |
| Construction of pLS169 library ($EntNfsB_{Lib}$) | Sequence encoding EntNfsB of *Enterobacter cloacae* | oligoLS363 | Rv - *BbsI* | TCTCTGAAGACTCGGTGCTGGCTACAATGAAGTGCCACGGCTGGGAGTTNNNNNNGGACGGGCTGTACTGC |
| Construction of pLS169 | Sequence encoding EntNfsB of *Enterobacter cloacae* | oligoLS366 | Fw - *BbsI* | CTCTGAAGACCAGTGGATGGCGAAGCAGGTTTACCTGAACGTCGG |

| | | | | |
|---|---|---|---|---|
| library (EntNfsB$_{Lib}$) | | | | |
| Construction of pLS169 library (EntNfsB$_{Lib}$) | Sequence encoding EntNfsB of *Enterobacter cloacae* | oligoLS364 | Fw - *BbsI* | CTCTGAAGACAGCACCGAGGAAGGAAAAGCGCGCGTGGCGAAGTCCGCTGCGGGCACCNNNGTGTTCAACGAACG |
| Construction of pLS169 library (EntNfsB$_{Lib}$) | Sequence encoding EntNfsB of *Enterobacter cloacae* | oligoLS365 | Rv - *BbsI* | TCTCTGAAGACATCCACTGGTCGTCATCTTTCAGATCCACGCGGTGCATGTCGGCNNNGTAGGTGCGGCC |
| Construction of pLS180 | Sequence encoding EntNfsB with C-terminal 6xHis-tag | oligoLS385 | Rv - *BbsI* 6xHis-tag | TCTCTGAAGACTCCTTAGTGGTGGTGGTGGTGGTGGCACTCGGTCACAATCGTGCTCAGC |
| Construction of pLS180 | Sequence encoding EntNfsB with C-terminal 6xHis-tag | oligoLS386 | Fw - *BbsI* | TCTCTGAAGACAACATGGATATCATTTCTGTCGCCCTG |
| Construction of pLS181 | Sequence encoding EntNfsB$_{S1}$ with C-terminal 6xHis-tag | oligoLS385 | Rv - *BbsI* 6xHis-tag | TCTCTGAAGACTCCTTAGTGGTGGTGGTGGTGGTGGCACTCGGTCACAATCGTGCTCAGC |
| Construction of pLS181 | Sequence encoding EntNfsB$_{S1}$ with C-terminal 6xHis-tag | oligoLS386 | Fw - *BbsI* | TCTCTGAAGACAACATGGATATCATTTCTGTCGCCCTG |
| Construction of pLS182 | Sequence encoding EntNfsB$_{S2}$ with C-terminal 6xHis-tag | oligoLS385 | Rv - *BbsI* 6xHis-tag | TCTCTGAAGACTCCTTAGTGGTGGTGGTGGTGGTGGCACTCGGTCACAATCGTGCTCAGC |
| Construction of pLS182 | Sequence encoding EntNfsB$_{S2}$ with C-terminal 6xHis-tag | oligoLS386 | Fw - *BbsI* | TCTCTGAAGACAACATGGATATCATTTCTGTCGCCCTG |
| Sequencing | pUC19 | M13 | Rv | CAGGAAACAGCTATGACC |
| Sequencing | pUC19 | M13 | Fw | TGTAAAACGACGGCCAGT |
| Sequencing | pET28a | T7 | Fw | TAATACGACTCACTATAGGG |
| Sequencing | pLS60 | oligoLS275 | Fw | CATCCTATGGAACTGCCTCG |
| Sequencing | pLS60 | oligoLS276 | Fw | GAAAGTGAATGTCAACGGCG |
| Sequencing | pLS60 | oligoLS277 | Fw | CATCGTTGCGACACACTTGGC |
| Sequencing | pLS60 | oligoLS278 | Fw | CTGCACCATGCCACTCACTG |
| Sequencing | pLS60 | oligoLS279 | Fw | CCGTACATGAAGCTTGGACAGG |

**Supplementary Table 5. Synthetic DNA sequences used in this project.** All synthetic genes were codon-adapted suitably for expression in *E. coli* and chemically synthesized (IDT or DNA2.0). Restriction sites are highlighted in green and start and stop codons in bold.

| Synthetic DNA | Sequence (5'- 3') | RBS and Restriction sites |
|---|---|---|
| **Sequence encoding 2,3-butanediol dehydrogenase BUDC of *Klebsiella pneumoniae*** | CCGTTCGCATGCCAATCTTAATCAA<br>ATCAGACAGAGAGAGTACAAT**ATG**A<br>AAAAAGTCGCACTTGTTACCGGCGC<br>CGGCCAGGGGATTGGTAAAGCTAT<br>CGCCCTTCGTCTGGTGAAGGATGG<br>ATTTGCCGTGGCCATTGCCGATTAT<br>AACGACACCACCGCCAAAGCGGTC<br>GCCTCCGAAATCAACCAGGCCGGC<br>GGCCGCGCCATGGCGGTGAAAGTG<br>GATGTCTCCGACCGCGATCAGGTG<br>TTTGCCGCCGTCGAACAGGCGCGC<br>AAAACGCTGGGCGGCTTCGACGTC<br>ATCGTCAACAACGCCGGCGTGGCG<br>CCGTCCACGCCGATCGAGTCCATT<br>ACCCCGGAGATTGTCGATAAAGTCT<br>ACAACATCAACGTTAAAGGGGTGAT<br>CTGGGGCATTCAGGCGGCGGTCGA<br>GGCCTTTAAGAAAGAGGGTCACGG<br>CGGGAAAATCATCAACGCCTGTTCC<br>CAGGCCGGCCACGTCGGCAACCCG<br>GAGCTGGCGGTATATAGCTCGAGT<br>AAATTCGCCGTACGCGGCTTAACCC<br>AGACCGCCGCTCGCGACCTCGCGC<br>CGCTGGGCATCACAGTCAACGGCT<br>ACTGCCCGGGGATTGTCAAAACGC<br>CAATGTGGGCCGAAATTGACCGCC<br>AGGTGTCCGAAGCCGCCGGTAAAC<br>CGCTGGGTTACGGTACCGCCGAGT<br>TCGCCAAACGCATCACCCTCGGCC<br>GCCTGTCCGAGCCGGAAGATGTCG<br>CCGCCTGCGTCTCCTATCTTGCCAG<br>CCCGGATTCTGATTATATGACCGGT<br>CAGTCATTGCTGATCGACGGCGGG<br>ATGGTGTTTAAC**TAA**GGATCCGCTG<br>AA | *SphI* (5'end) *BamHI* (3'end)<br><br>Synthetic RBS sequence:<br>CAATCTTAATCAAATCAGACAGAGA<br>GAGTACAAT |
| **Sequence encoding NAD-dependent alcohol dehydrogenase TADH of *Thermus* sp. ATN-1** | CCGTTCGCATGCAGGAGGTACGAA<br>CAC**ATG**AAGGGGTTCGCAATGCTG<br>TCTATTGGAAAAGTTGGCTGGATTG<br>AAAAGGAGAAGCCAGCGCCAGGGC<br>CTTTCGACGCAATTGTTCGCCCTTT<br>GGCAGTCGCACCTTGCACGTCTGA<br>CATCCACACCGTTTTCGAAGGAGCC<br>ATTGGTGAACGTCATAACATGATCT<br>TGGGACACGAAGCGGTAGGTGAGG<br>TTGTAGAGGTCGGTTCTGAAGTTAA<br>GGACTTTAAACCTGGAGACCGCGT<br>GGTGGTGCCCGCGATTACGCCTGA<br>CTGGCGTACTTCAGAGGTCCAACG<br>TGGATATCACCAACATAGCGGCGG<br>TATGCTGGCGGGTTGGAAGTTCTC<br>CAATGTGAAGGACGGTGTTTTCGGA<br>GAATTCTTCCATGTTAATGACGCCG<br>ACATGAATTTGGCGCACCTTCCGAA<br>GGAGATTCCGTTAGAAGCCGCGGT<br>AATGATCCCCGACATGATGACCACC<br>GGCTTTCATGGAGCGGAGCTGGCG<br>GACATCGAGTTGGGCGCTACCGTG<br>GCTGTACTTGGCATCGGTCCTGTC<br>GGTCTGATGGCGGTGGCAGGGGC<br>CAAGTTGCGTGGTGCAGGACGTAT<br>CATTGCTGTTGGTTCTCGTCCAGTC<br>TGTGTCGACGCTGCCAAGTATTACG<br>GAGCAACCGACATCGTGAACTATAA<br>GGATGGGCCAATTGAGTCACAGATT<br>ATGAACCTTACAGAAGGGAAGGGA<br>GTTGATGCAGCTATTATTGCAGGCG<br>GGAATGCGGATATCATGGCGACAG<br>CCGTCAAGATCGTGAAGCCCGGTG | *SphI* (5'end) *BamHI* (3'end)<br><br>Shine-Dalgarno RBS:<br>AGGAGGTACGAACAC |

| | | |
|---|---|---|
| | GAACTATTGCTAATGTGAATTACTTT<br>GGTGAGGGAGAAGTTTTGCCGGTG<br>CCTCGCCTGGAATGGGGTTGTGGG<br>ATGGCCCACAAAACGATCAAGGGA<br>GGTCTGTGTCCAGGGGGACGTCTG<br>CGCATGGAACGCTTGATTGACCTTG<br>TCTTTTACAAACGTGTGGACCCGAG<br>TAAATTGGTCACACACGTATTCCGT<br>GGCTTTGATAACATTGAAAAGGCGT<br>TCATGTTGATGAAGGATAAACCAAA<br>GGACCTTATCAAACCAGTGGTTATT<br>CTGGCT**TAA**GGATCCTTCAGC | |
| **Sequence encoding NADP-dependent isopropanol dehydrogenase CBADH of *Clostridium beijerinckii*** | GTTCGCATGCATTCGGATCTATACA<br>GATAAGGAGAAAGAGATGAAAGGC<br>TTTGCC**ATG**CTGGGTATTAACAAAT<br>TAGGATGGATTGAAAAAGAACGCCC<br>CGTCGCGGGTTCCTATGATGCGATT<br>GTACGACCCTTAGCCGTTTCCCCGT<br>GCACTAGCGATATTCATACAGTATT<br>TGAAGGGGCTCTCGGCGATCGAAA<br>GAATATGATTTTAGGCCATGAAGCC<br>GTTGGCGAAGTCGTTGAAGTGGGC<br>TCCGAAGTGAAAGATTTCAAACCGG<br>GTGACCGTGTCATCGTGCCCTGTA<br>CTACCCCAGATTGGCGCTCTCTGG<br>AGGTTCAAGCTGGTTTTCAACAACA<br>TAGTAATGGTATGTTGGCCGGCTG<br>GAAGTTTTCCAACTTCAAAGATGGA<br>GTATTTGGGGAGTATTTTCATGTGA<br>ACGATGCGGATATGAATTTGGCCAT<br>CCTGCCAAAAGACATGCCCTTGGA<br>GAATGCTGTAATGATCACCGATATG<br>ATGACCACCGGATTTCATGGGGCC<br>GAGTTGGCCGATATCCAGATGGGT<br>AGTTCTGTCGTTGTGATTGGTATCG<br>GGGCAGTTGGGTTAATGGGAATTG<br>CTGGGGCCAAATTACGCGGAGCAG<br>GTCGGATTATTGGTGTCGGCAGTC<br>GGCCTATTTGCGTTGAGGCCGCCA<br>AGTTCTACGGCGCGACCGACATTCT<br>GAATTACAAAAATGGCCATATTGTG<br>GACCAGGTAATGAAGCTAACCAATG<br>GGAAAGGCGTGGACCGTGTGATTA<br>TGGCTGGAGGTGGGAGTGAAACAC<br>TGAGCCAAGCAGTGAGCATGGTGA<br>AACCTGGGGGAATTATCAGCAATAT<br>CAACTATCACGGCTCTGGTGACGCT<br>TTGTTAATTCCCCGCGTGGAATGGG<br>GATGTGGCATGGCGCACAAGACGA<br>TCAAAGGCGGTTTGTGTCCCGGAG<br>GCCGTTTACGGGCCGAAATGCTAC<br>GGGATATGGTGGTGTACAACCGTG<br>TGGATTTGTCCAAGCTGGTGACTCA<br>CGTTTATCACGGTTTTGACCATATT<br>GAAGAAGCCTTGCTACTCATGAAAG<br>ATAAACCTAAAGATCTCATTAAGGC<br>CGTAGTTATCCTC**TAA**TAGTGAGGA<br>TCCATGGAAG | *SphI* (5'end) *BamHI* (3'end)<br><br>Synthetic RBS sequence:<br>ATTCGGATCTATACAGATAAGGAGA<br>AAGAGATGAAAGGCTTTGCC |
| **Sequence encoding NADP-dependent isopropanol dehydrogenase TBADH of *Thermoanaerobacter brockii*** | CCGTTCGCATGCAGGAGGTACGAA<br>CAC**ATG**AAGGGGTTCGCAATGCTG<br>TCTATTGGAAAAGTTGGCTGGATTG<br>AAAAGGAGAAGCCAGCGCCAGGGC<br>CTTTCGACGCAATTGTTCGCCCTTT<br>GGCAGTCGCACCTTGCACGTCTGA<br>CATCCACACCGTTTTCGAAGGAGCC<br>ATTGGTGAACGTCATAACATGATCT<br>TGGGACACGAAGCGGTAGGTGAGG<br>TTGTAGAGGTCGGTTCTGAAGTTAA<br>GGACTTTAAACCTGGAGACCGCGT<br>GGTGGTGCCCGCGATTACGCCTGA<br>CTGGCGTACTTCAGAGGTCCAACG<br>TGGATATCACCAACATAGCGGCGG<br>TATGCTGGCGGGTTGGAAGTTCTC<br>CAATGTGAAGGACGGTGTTTTCGGA<br>GAATTCTTCCATGTTAATGACGCCG | *SphI* (5'end) *BamHI* (3'end)<br><br>Shine-Dalgarno RBS:<br>AGGAGGTACGAACAC |

| | | |
|---|---|---|
| | ACATGAATTTGGCGCACCTTCCGAA GGAGATTCCGTTAGAAGCCGCGGT AATGATCCCCGACATGATGACCACC GGCTTTCATGGAGCGGAGCTGGCG GACATCGAGTTGGGCGCTACCGTG GCTGTACTTGGCATCGGTCCTGTC GGTCTGATGGCGGTGGCAGGGGC CAAGTTGCGTGGTGCAGGACGTAT CATTGCTGTTGGTTCTCGTCCAGTC TGTGTCGACGCTGCCAAGTATTACG GAGCAACCGACATCGTGAACTATAA GGATGGGCCAATTGAGTCACAGATT ATGAACCTTACAGAAGGGAAGGGA GTTGATGCAGCTATTATTGCAGGCG GGAATGCGGATATCATGGCGACAG CCGTCAAGATCGTGAAGCCCGGTG GAACTATTGCTAATGTGAATTACTTT GGTGAGGGAGAAGTTTTGCCGGTG CCTCGCCTGGAATGGGGTTGTGGG ATGGCCCACAAAACGATCAAGGGA GGTCTGTGTCCAGGGGGACGTCTG CGCATGGAACGCTTGATTGACCTTG TCTTTTACAAACGTGTGGACCCGAG TAAATTGGTCACACACGTATTCCGT GGCTTTGATAACATTGAAAAGGCGT TCATGTTGATGAAGGATAAACCAAA GGACCTTATCAAACCAGTGGTTATT CTGGCT**TAA**GGATCCTTCAGC | |
| **Sequence encoding NADP-dependent (R)-selective imine reductase MsIRED of *Myxococcus stipitatus*** | CCGTTCGCATGCAGGAGGTACGAA CAC**ATG**AAACCGACCCTGACCGTTA TTGGCGCTGGCCGTATGGGCTCCG CACTGATTAAAGCATTCCTGCAATC TGGCTACACGACCACGGTGTGGAA CCGTACCAAAGCCAAAAGCGAACC GCTGGCAAAACTGGGCGCACATCT GGCTGATACGGTGCGTGACGCCGT TAAACGCAGCGATATTATCGTGGTT AATGTGCTGGATTATGACACCTCTG ATCAGCTGCTGCGCCAAGACGAAG TGACGCGTGAACTGCGCGGCAAAC TGCTGGTTCAGCTGACCAGCGGTT CTCCGGCACTGGCTCGTGAACAGG AAACGTGGGCGCGCCAACATGGCA TTGATTATCTGGACGGTGCGATCAT GGCCACCCCGGATTTTATTGGCCA GGCAGAATGCGCTCTGCTGTACAG TGGTTCCGCGGCCCTGTTCGAAAA ACACCGTGCTGTCCTGAATGTGCTG GGCGGTGCCACCAGCCATGTCGGC GAAGATGTTGGTCATGCCTCAGCAC TGGACAGCGCCCTGCTGTTTCAGAT GTGGGGCACCCTGTTCGGTACGCT GCAAGCACTGGCTATTTCTCGCGCA GAAGGCATCCCGCTGGAAAAAACC ACGGCGTTTATCAAACTGACCGAAC CGGTCACCCAGGGTGCCGTTGCAG ATGTCCTGACCCGTGTTCAGCAAAA TCGCCTGACCGCAGACGCTCAGAC GCTGGCAAGTCTGGAAGCTCATAA CGTGGCGTTCCAACACCTGCTGGC CCTGTGTGAAGAACGTAATATCCAT CGCGGTGTTGCGGATGCCATGTAC TCCGTTATTCGTGAAGCGGTCAAAG CCGGCCACGGTAAAGATGACTTTG CAATTCTGACCCGCTTCCTGAAA**TA A**GGATCCTTCAGC | *SphI* (5'end) *BamHI* (3'end)<br><br>Shine-Dalgarno RBS: AGGAGGTACGAACAC |
| **Sequence encoding oxygen-insensitive NAD(P)-dependent nitroreductase EntNfsB (NfsB) of *Enterobacter cloacae*** | CATCTGAAGACAAC**ATG**GATATCAT TTCTGTCGCCCTGAAACGCCACTCT ACCAAGGCGTTCGACGCAAGCAAA AAACTGACCGCGGAAGAAGCGGAA AAAATCAAAACCCTGCTGCAGTACA GCCCGTCCAGCACCAACTCCCAGC CGTGGCACTTCATTGTAGCCAGCAC CGAGGAAGGAAAAGCGCGCGTGGC GAAGTCCGCTGCGGGCACCTATGT | *BbsI* (5'end)<br>*BbsI* (3'end) |

| | | |
|---|---|---|
| | GTTCAACGAACGCAAAATGCTGGAT<br>GCTTCCCACGTGGTGGTGTTCTGC<br>GCGAAAACCGCGATGGATGACGCC<br>TGGCTGGAGCGCGTCGTGGATCAG<br>GAAGAGGCCGATGGCCGTTTCAAC<br>ACGCCGGAAGCCAAAGCCGCAAAC<br>CATAAGGGCCGCACCTACTTCGCC<br>GACATGCACCGCGTGGATCTGAAA<br>GATGACGACCAGTGGATGGCGAAG<br>CAGGTTTACCTGAACGTCGGCAACT<br>TCCTGCTGGGCGTGGGCGCGATGG<br>GTCTGGACGCGGTACCAATTGAAG<br>GTTTCGACGCCGCTATTCTCGACGA<br>AGAGTTTGGCCTGAAAGAGAAAGG<br>CTTCACCAGCCTGGTGGTGGTACC<br>GGTTGGGCACCACAGCGTGGAAGA<br>TTTCAACGCCACGCTGCCGAAATCT<br>CGCCTGCCGCTGAGCACGATTGTG<br>ACCGAGTGC**TAA**GGAGTCTTCAGA<br>GA | |

**Supplementary Table 6. Sequence of saturated positions for 10 random variants of the CBADH library and the selected variant.**

| Enzyme / variant | Position | | | |
|---|---|---|---|---|
| | 198 | 199 | 200 | 218 |
| **WT** | GGC | AGT | CGG | TAC |
| **R1** | TCC | CGG | ACC | TTG |
| **R2** | TTA | TTC | TAA | Deletion of 210 bp |
| **R3** | TTC | CGT | ATA | CGG |
| **R4** | TCA | GCT | GTA | TAG |
| **R5** | AAT | GTG | ACA | GGG |
| **R6** | CTA | GCG | AAC | GGG |
| **R7** | CAT | CTC | CAA | ACC |
| **R8** | AAC | TCC | TCA | GTG |
| **R9** | AAA | CCA | TCA | GTT |
| **R10** | GCC | CCC | AGG | CGT |
| **CBADHs** | GAC | TAT | AGA | CCG |

**Supplementary Table 7. Sequence of saturated positions for 10 random variants of the TBADH library and selected variants.** $TBADH_{S2}$ contained a duplication of residues 191 to 241, in addition to substitutions in the targeted residues both in the positions of the original sequence and in the corresponding positions of the duplication. 198', 199', 200' and 218' denote positions in the duplication equivalent to the original residues 198, 199, 200 and 218. These positions only exist in $TBADH_{S2}$.

| **Enzyme / variant** | **Position** | | | | | | | |
|---|---|---|---|---|---|---|---|---|
| | 198 | 199 | 200 | 218 | 198' | 199' | 200' | 218' |
| **WT** | GGT | TCT | CGT | TAT | - | - | - | - |
| **R1** | CTT | TCC | CGC | TTA | - | - | - | - |
| **R2** | TGA | CGC | CGA | GTT | - | - | - | - |
| **R3** | CTC | TCC | CGC | GTG | - | - | - | - |
| **R4** | AAT | CAA | AAG | AGG | - | - | - | - |
| **R5** | TTA | ACA | CGA | CGC | - | - | - | - |
| **R6** | AGC | ACC | CGA | CGA | - | - | - | - |
| **R7** | AAC | TGA | ACT | GTA | - | - | - | - |
| **R8** | CCC | CAG | AGG | AGA | - | - | - | - |
| **R9** | Insertion 153 bp GCA | TCA | CGT | TAC | - | - | - | - |
| **R10** | GAC | TAT | AGA | CCG | - | - | - | - |
| **$TBADH_{S1}$** | TCA | AAA | CGG | GTA | - | - | - | - |
| **$TBADH_{S2}$** | CAC | CGC | GCC | ATG | GCA | TCA | AAA | TAC |

**Supplementary Table 8. Sequence of saturated positions for 10 random variants of the MsIRED library and the selected variant.**

| Enzyme / variant | Position | | | | | |
|---|---|---|---|---|---|---|
| | 32 | 33 | 34 | 37 | 67 | 71 |
| **WT** | AAC | CGT | ACC | AAA | ATC | ACC |
| **R1** | GGT | GGT | GAT | GGG | | |
| **R2** | GGT | GGG | GGG | GGG | | |
| **R3** | GTG | TCT | GCT | GGG | | |
| **R4** | CTT | CGT | ACT | TTT | | |
| **R5** | CAT | GAT | GCG | CAG | | |
| **R6** | TTG | CTT | TAT | GTG | | |
| **R7** | CTA | ATT | GTT | ACT | | |
| **R8** | CCG | AAG | TAT | TGT | | |
| **R9** | ATT | GTT | TAG | ATG | | |
| **R10** | GAG | CGG | GTG | TTG | | |
| **$MSIRED_C$** | GAA | TAT | GAA | CGT | ATT | GTT |
| **$MSIRED_S$** | GAG | GTG | CGG | CGG | | |

**Supplementary Table 9. Sequence of saturated positions for 10 random variants of the EntNfsB library and the selected variants.**

| Enzyme / variant | Position | | | |
|---|---|---|---|---|
| | 40 | 41 | 68 | 124 |
| **WT** | AGC | ACC | TAT | TTC |
| **R1** | TTA | TTA | GTG | CAA |
| **R2** | AAC | GTA | TAG | CCC |
| **R3** | TCA | GTA | AAT | GTA |
| **R4** | TAC | ATA | CCT | ACC |
| **R5** | GTA | TTA | TGT | TGC |
| **R6** | AGC | TTC | AAG | TTA |
| **R7** | TCC | TTC | TTT | CAA |
| **R8** | CGA | ATA | TAG | TGC |
| **R9** | CTA | TAA | TCG | CCC |
| **R10** | CAA | AAC | ATG | CCA |
| **EntNfsB$_{S1}$** | GCA | ATA | TAT | GCA |
| **EntNfsB$_{S2}$** | TCA | CTA | CTT | CTC |

**Supplementary Table 10. Conditions for anaerobic growth experiments.**

| Mutant strain | Transformed plasmid/library | Antibiotic | Added external substrate | Generated reduced product |
|---|---|---|---|---|
| **AL** | pUC19 | Ampicillin | As required for the complementation experiment | - |
| **AL** | pLS1 | Ampicillin | - | - |
| **AL** | pLS2 | Ampicillin | 15 mM acetoin | 2,3-butanediol |
| **AL** | pLS3 | Ampicillin | 15 mM acetoin | 2,3-butanediol |
| **AL** | pLS6 | Ampicillin | 15 mM acetone | Isopropanol |
| **AL** | pLS10 (library) | Ampicillin | 15 mM acetone | Isopropanol |
| **AL** | pLS10_3 | Ampicillin | 15 mM acetone | Isopropanol |
| **AL** | pLS12 | Ampicillin | 10 mM cyclohexanone or 3-methylcyclohexanone | Cyclohexanol or 3-methylcyclohexanol |
| **AL** | pLv2 | Kanamycin | - | - |
| **AL** | pLS60 | Kanamycin | - | Isopropanol |
| **ALPS** | pLS69 | Ampicillin | 15 mM acetone | Isopropanol |
| **ALPS** | pLS73 (library) | Ampicillin | 15 mM acetone | Isopropanol |
| **ALPS** | pLS73_1 | Ampicillin | 15 mM acetone | Isopropanol |
| **ALPS** | pLS73_2 | Ampicillin | 15 mM acetone | Isopropanol |
| **AL** | pLS130 | Ampicillin | 15 mM 2-methyl-1-pyrroline | 2-methylpyrrolidine |
| **AL** | pLS131 | Ampicillin | 15 mM 2-methyl-1-pyrroline | 2-methylpyrrolidine |
| **AL** | pLS133 (library) | Ampicillin | 15 mM 2-methyl-1-pyrroline | 2-methylpyrrolidine |
| **AL** | pLS133_1 | Ampicillin | 15 mM 2-methyl-1-pyrroline | 2-methylpyrrolidine |
| **AL** | pLS168 | Ampicillin | 2.5 mM 4-nitrobenzoic acid | 4-aminobenzoic acid |
| **AL** | pLS168 | Ampicillin | 8 mM 2-nitrobenzoic acid | 2-aminobenzoic acid |
| **AL** | pLS168 | Ampicillin | 2.5 mM 4-nitrobenzyl alcohol | 4-aminobenzyl alcohol |
| **AL** | pLS169 (library) | Ampicillin | 8 mM 2-nitrobenzoic acid | 2-aminobenzoic acid |
| **AL** | pLS169_1 | Ampicillin | 8 mM 2-nitrobenzoic acid | 2-aminobenzoic acid |
| **AL** | pLS169 (library) | Ampicillin | 2.5 mM 4-nitrobenzyl alcohol | 4-aminobenzyl alcohol |
| **AL** | pLS169_3 | Ampicillin | 2.5 mM 4-nitrobenzyl alcohol | 4-aminobenzyl alcohol |

**Supplementary Table 11. Characteristic $^1$H-NMR signals used to quantify each compound of interest.** The multiplicity of each signal (s – singlet, d – doublet, t – triplet, q – quartet, m – multiplet) and the number of contributing protons is shown between brackets.

| Metabolite | δ for characteristic signal of the metabolite (ppm) |
|---|---|
| **Ethanol** | 1.19 (t,3) |
| **Lactate** | 1.32 (d,3) |
| **Succinate** | 2.41 (s,4) |
| **Acetate** | 1.92 (s,3) |
| **Formate** | 8.46 (s,1) |
| **Acetoin** | 1.38 (d, 3) |
| **2,3-butanediol** | 1.15 (d,6) |
| **Cyclohexanone** | 2.40 (t,4) |
| **Cyclohexanol** | 1.72 (m, 2) |
| **3-methylcyclohexanone** | 1.01 (d,3) |
| **3-methylcyclohexanol** | 0.88 (d,3) |
| **Acetone** | 2.24 (s,6) |
| **Isopropanol** | 1.18 (d,6) |
| **Propionate** | 2.19 (q, 2) |
| **2-methyl-1-pyrroline** | 2.42 (s,3) |
| **2-methylpyrrolidine** | 1.38 (d,3) |
| **4-nitrobenzoic acid** | 8.01 (d,2) |
| **4-aminobenzoic acid** | 7.73 (d,2) |
| **2-nitrobenzoic acid** | 8.11 (d,1) |
| **2-aminobenzoic acid** | 7.30 (t,1) |
| **4-nitrobenzyl alcohol** | 8.27 (d,2) |
| **4-aminobenzyl alcohol** | 7.23 (d,2) |

**Supplementary Table 12. Conditions for enzymatic activity assays.**

| Enzyme | Substrate | Cofactor | Enzyme concentration | Absorbance (nm) |
|---|---|---|---|---|
| **$CBADH_{WT}$** | Isopropanol | 1 mM $NADP^+$ | 110 nM | 340 |
| **$CBADH_{WT}$** | Isopropanol | 10 mM $NAD^+$ | 110 nM | 340 |
| **$CBADH_S$** | Isopropanol | 1 mM $NADP^+$ | 110 nM | 340 |
| **$CBADH_S$** | Isopropanol | 10 mM $NAD^+$ | 110 nM | 340 |
| **$TBADH_{WT}$** | Isopropanol | 1.2 mM $NADP^+$ | 110.8 nM | 340 |
| **$TBADH_{WT}$** | Isopropanol | 10 mM $NAD^+$ | 110.8 nM | 340 |
| **$TBADH_{S1}$** | Isopropanol | 1.2 mM $NADP^+$ | 110 nM | 340 |
| **$TBADH_{S1}$** | Isopropanol | 10 mM $NAD^+$ | 110 nM | 340 |
| **$TBADH_{S2}$** | Isopropanol | 2.35 mM $NADP^+$ | 51.55 nM | 340 |
| **$TBADH_{S2}$** | Isopropanol | 2.35 mM $NAD^+$ | 51.55 nM | 340 |
| **$MsIRED_{WT}$** | 2-methyl-1-pyrroline | 0.25 mM NADPH | 1.2 µM | 340 |
| **$MsIRED_{WT}$** | 2-methyl-1-pyrroline | 0.25 mM NADH | 1.2 µM | 340 |
| **$MsIRED_C$** | 2-methyl-1-pyrroline | 0.25 mM NADH | 1.26 µM | 340 |
| **$MsIRED_S$** | 2-methyl-1-pyrroline | 0.25 mM NADH | 1.25 µM | 340 |
| **$EntNfsB_{WT}$** | 4-nitrobenzoic acid | 0.3 mM NADH | 60.8 nM | 370 |
| **$EntNfsB_{WT}$** | 2-nitrobenzoic acid | 0.3 mM NADH | 60.8 nM | 370 |
| **$EntNfsB_{WT}$** | 4-nitrobenzyl alcohol | 0.3 mM NADH | 26.3 nM | 370 |
| **$EntNfsB_{S1}$** | 4-nitrobenzoic acid | 0.3 mM NADH | 45 nM | 370 |
| **$EntNfsB_{S1}$** | 2-nitrobenzoic acid | 0.3 mM NADH | 45 nM | 370 |
| **$EntNfsB_{S2}$** | 4-nitrobenzyl alcohol | 0.3 mM NADH | 26.3 nM | 370 |

**Supplementary Table 13. Data collection parameters and refinement statistics for the crystal structures of CBADHs and TBADHS1.**

| | CBADHs variant | TBADHS1 variant |
|---|---|---|
| Beamline | Diamond I03 | Diamond I04 |
| PDB code | 6SCH | 6SDM |
| Wavelength | 0.9762 | 0.9795 |
| Resolution range | 59.36 - 2.199 (2.278 - 2.199) | 71.68 - 2.85 (2.952 - 2.85) |
| Space group | P 1 21 1 | P 21 21 21 |
| Unit cell | 75.8115 99.5695 114.089 90 102.768 90 | 79.1234 123.946 169.24 90 90 90 |
| Total reflections | 265743 (16967) | 258210 (26258) |
| Unique reflections | 82558 (7335) | 39642 (3923) |
| Multiplicity | 3.2 (2.3) | 6.5 (6.7) |
| Completeness (%) | 98.45 (87.66) | 99.87 (99.62) |
| Mean I/sigma(I) | 7.57 (0.71) | 6.92 (1.11) |
| Wilson B-factor | 34.89 | 69.95 |
| R-merge | 0.09525 (1.064) | 0.1918 (1.671) |
| R-meas | 0.114 (1.345) | 0.2086 (1.811) |
| R-pim | 0.06202 (0.8095) | 0.08117 (0.6942) |
| CC1/2 | 0.996 (0.345) | 0.991 (0.554) |
| CC* | 0.999 (0.716) | 0.998 (0.844) |
| Reflections used in refinement | 82766 (7315) | 39597 (3913) |
| Reflections used for R-free | 4037 (357) | 2015 (182) |
| R-work | 0.1639 (0.2548) | 0.1746 (0.3201) |
| R-free | 0.2068 (0.2927) | 0.2390 (0.3647) |
| CC(work) | 0.970 (0.619) | 0.955 (0.750) |
| CC(free) | 0.960 (0.587) | 0.940 (0.651) |
| Number of non-hydrogen atoms | 11354 | 10622 |
| macromolecules | 10666 | 10618 |
| ligands | 332 | 4 |
| solvent | 356 | 0 |
| Protein residues | 1417 | 1416 |
| RMS(bonds) | 0.007 | 0.009 |
| RMS(angles) | 0.93 | 1.06 |
| Ramachandran favored (%) | 95.87 | 95.10 |
| Ramachandran allowed (%) | 4.13 | 4.69 |
| Ramachandran outliers (%) | 0.00 | 0.21 |
| Rotamer outliers (%) | 0.97 | 0.27 |
| Clashscore | 5.11 | 4.48 |
| Average B-factor | 45.12 | 77.08 |
| macromolecules | 44.13 | 77.04 |
| ligands | 78.13 | 174.49 |
| solvent | 43.81 | - |
| Number of TLS groups | 18 | 15 |
